# Development and Deployment of the OpenMRS-Ebola Electronic Health Record System for an Ebola Treatment Center in Sierra Leone

**DOI:** 10.2196/jmir.7881

**Published:** 2017-08-21

**Authors:** Shefali Oza, Darius Jazayeri, Jonathan M Teich, Ellen Ball, Patricia Alexandra Nankubuge, Job Rwebembera, Kevin Wing, Alieu Amara Sesay, Andrew S Kanter, Glauber D Ramos, David Walton, Rachael Cummings, Francesco Checchi, Hamish S Fraser

**Affiliations:** ^1^ Faculty of Epidemiology and Population Health London School of Hygiene and Tropical Medicine London United Kingdom; ^2^ Save the Children International Kerry Town Sierra Leone; ^3^ OpenMRS Inc Indianapolis, IN United States; ^4^ ThoughtWorks Kampala Uganda; ^5^ Brigham and Women’s Hospital Boston, MA United States; ^6^ Partners In Health Boston, MA United States; ^7^ Columbia University New York City, NY United States; ^8^ Save the Children UK London United Kingdom; ^9^ University of Leeds Leeds United Kingdom

**Keywords:** Ebola virus disease, electronic health records, eHealth, health information systems, disease outbreaks, disasters, West Africa, Sierra Leone

## Abstract

**Background:**

Stringent infection control requirements at Ebola treatment centers (ETCs), which are specialized facilities for isolating and treating Ebola patients, create substantial challenges for recording and reviewing patient information. During the 2014-2016 West African Ebola epidemic, paper-based data collection systems at ETCs compromised the quality, quantity, and confidentiality of patient data. Electronic health record (EHR) systems have the potential to address such problems, with benefits for patient care, surveillance, and research. However, no suitable software was available for deployment when large-scale ETCs opened as the epidemic escalated in 2014.

**Objective:**

We present our work on rapidly developing and deploying OpenMRS-Ebola, an EHR system for the Kerry Town ETC in Sierra Leone. We describe our experience, lessons learned, and recommendations for future health emergencies.

**Methods:**

We used the OpenMRS platform and Agile software development approaches to build OpenMRS-Ebola. Key features of our work included daily communications between the development team and ground-based operations team, iterative processes, and phased development and implementation. We made design decisions based on the restrictions of the ETC environment and regular user feedback. To evaluate the system, we conducted predeployment user questionnaires and compared the EHR records with duplicate paper records.

**Results:**

We successfully built OpenMRS-Ebola, a modular stand-alone EHR system with a tablet-based application for infectious patient wards and a desktop-based application for noninfectious areas. OpenMRS-Ebola supports patient tracking (registration, bed allocation, and discharge); recording of vital signs and symptoms; medication and intravenous fluid ordering and monitoring; laboratory results; clinician notes; and data export. It displays relevant patient information to clinicians in infectious and noninfectious zones. We implemented phase 1 (patient tracking; drug ordering and monitoring) after 2.5 months of full-time development. OpenMRS-Ebola was used for 112 patient registrations, 569 prescription orders, and 971 medication administration recordings. We were unable to fully implement phases 2 and 3 as the ETC closed because of a decrease in new Ebola cases. The phase 1 evaluation suggested that OpenMRS-Ebola worked well in the context of the rollout, and the user feedback was positive.

**Conclusions:**

To our knowledge, OpenMRS-Ebola is the most comprehensive adaptable clinical EHR built for a low-resource setting health emergency. It is designed to address the main challenges of data collection in highly infectious environments that require robust infection prevention and control measures and it is interoperable with other electronic health systems. Although we built and deployed OpenMRS-Ebola more rapidly than typical software, our work highlights the challenges of having to develop an appropriate system during an emergency rather than being able to rapidly adapt an existing one. Lessons learned from this and previous emergencies should be used to ensure that a set of well-designed, easy-to-use, pretested health software is ready for quick deployment in future.

## Introduction

### Background

The 2014-2016 West African Ebola epidemic, with more than 28,000 infected and 11,000 deaths, overwhelmed health systems in three of the world’s most impoverished countries [1]. Existing health facilities in the affected areas did not have the necessary capacity, staff, and infection control capabilities to adequately cope with this outbreak [2]. Untreated Ebola patients not only have a high mortality rate but also remain a serious infection risk to their communities [3]. In this context, large-scale Ebola treatment centers (ETCs) emerged as emergency health facilities that could be set up quickly to isolate and treat seriously ill patients while providing the rigorous infection control needed to protect staff from the Ebola virus.

ETCs are specialized facilities that must provide efficient care for suspected and confirmed Ebola patients while minimizing the risk of infection to staff and other patients. This presents several challenges [4], including overheating, impaired visibility, and poor dexterity caused by working in highly restrictive personal protective equipment (PPE); limited time for direct patient contact; and the inability to move material—including paper medical records—from highly infectious patient areas (red zone) to low-risk nonpatient areas (green zone).

### Data Challenges in Ebola Treatment Centers

A key component of ETC patient management is the collection, transmission, use, and analysis of clinical data. Although the simplicity of paper records makes them a practical and appealing option for health information recording during an emergency, they can also be inflexible and difficult to share. This is particularly true when clinical data are used by multiple teams or by public health authorities and researchers in different locations, as well as where physical and contagion boundaries restrict the use of paper. In the case of Ebola, highly restrictive infection controls in ETCs created problems across a range of standard hospital operations when using paper records such as recording sufficient information legibly on patient charts while wearing PPE, accessing bedside charts, and communicating patient information between the zones.

One of the main data challenges during this outbreak when using paper-based data collection systems involved extracting patient data from the red zone. Methods for doing this imposed severe compromises on the quality, quantity, and confidentiality of patient data that were collected and transferred. Communication methods used by organizations have included shouting or radioing of information, such as prescriptions, to the green zone; photographing red zone whiteboards with identifiable patient information; and time-consuming scanning of often illegible or damaged records over a local wireless network [5,6]. Overall, conventional paper-based data collection and review is difficult in large-scale ETCs and limits the ability to gather urgently needed data for care, surveillance, and research. Due to this, it was recognized that electronic health record (EHR) systems could potentially offer substantial benefits over conventional paper-based records in an ETC. Yet EHRs, which are standard in many health care settings, are still rarely used effectively in health emergencies [7]. This is especially true for emergencies in low-resource settings.

### Electronic Patient Records for Ebola

As ETCs began opening in West Africa in 2014, there was no EHR software that could be rapidly deployed in these settings. Most EHR systems are designed for the needs of high-income health facilities that have reliable power, network infrastructure, and technical support. EHR systems for low-resource settings are increasingly common but to date have had limited use in hospital settings, especially for intensive care [8,9]. A district and national health data management system, Health Information System Programme’s district health information software (DHIS) 2, was available in West Africa at the time of the Ebola outbreak but is designed mainly for aggregate patient data instead of the individual-level records needed for patient care in an ETC [10]. Other systems, including a number of commercial EHR systems used in Africa, lacked the flexibility and rapid adaptability needed for deployment during this outbreak. Additionally, ETCs have substantially different workflows and information requirements from other acute and intensive care environments. Finally, an EHR must be easy to learn, as well as quick and simple to use in the uncomfortable, time-limited, dexterity- and vision-limited, strictly infection-controlled environment of the ETC.

OpenMRS is an open-source, modular EHR platform [11] for building patient medical record applications. First deployed in Kenya, Rwanda, and South Africa in 2006 [12] to support care of patients with human immunodeficiency virus (HIV) and tuberculosis (TB), it is now used to manage primary care and a range of diseases in more than 60 low- and middle-income countries (LMICs) [13,14]. It did not have a suitable user-friendly tablet interface needed for red zone patient data collection; however, the overall adaptability of OpenMRS, alongside an active community of programmers and users, made it a promising choice as an EHR platform for ETCs.

### Study Aims

In this paper, we describe our work on rapidly developing OpenMRS-Ebola, an open-source Ebola EHR system that was implemented in 2015 at Save the Children’s Kerry Town ETC in Sierra Leone. We describe our experiences, lessons learned, and recommendations for design and implementation of EHRs in future health emergencies.

## Methods

### Setting

The 80-bed Kerry Town ETC, based in the Western Area Rural district of Sierra Leone, operated from November 5, 2014 to March 31, 2015. Save the Children International (SCI) ran the site in collaboration with the Sierra Leone and United Kingdom governments. The ETC had wards for suspected and confirmed Ebola patients in the red zone and a range of operational rooms in the green zone, including a pharmacy, clinician station, and offices. The site employed over 100 clinicians, mostly from Sierra Leone and Cuba, with some non-Cuban international staff on rotation. Most patients came from nearby districts. The Kerry Town ETC had reliable power from 2 large generators. For connectivity, the site had a wireless local area network (WLAN) and a Ku-band very small aperture terminal (VSAT) system for Internet. The site also had backup power supply (uninterruptible power supply [UPS]) devices for the server, network routers, and other computing hardware.

### EHR Platform

OpenMRS is a highly flexible and configurable EHR platform [11]. It has a core system for managing log-ins, user accounts with security and privileges, data storage, and data retrieval. Plugin software modules extend the basic core system with custom functionality. This provides flexibility for different environments, disease types, and clinical workflows. The OpenMRS data model is built around a concept dictionary that structures and codes nearly all patient data and is mapped to standard medical terminology such as systematized nomenclature of medicine -- clinical terms (SNOMED CT) and International Statistical Classification of Diseases and Related Health Problems, Tenth Revision (ICD-10; [12]). OpenMRS is also compliant with health-level 7 (HL7) version 2.x and fast healthcare interoperability resources (FHIR) standards for data exchange [12,15]. It is interoperable with other electronic health (eHealth) systems such as DHIS 2, HL7-compliant laboratory information management systems, and several mobile health (mHealth) platforms, including ODK, CommCare, and Sana [16]. Recently, two new user interface (UI) and application tool kits have been developed to better support point-of-care use by clinicians and other staff [17]. Previous OpenMRS-related work in Malawi [18] and Rwanda [19] used touch screen computers. However, these were not designed for modern tablet devices or for the latest versions of the OpenMRS platform and would have required extensive adaptation to meet our red zone design needs.

We chose to use the latest available version of OpenMRS (v1.10.3) because it already had most of the functionality needed for an ETC EHR, it was open source and known to function well in challenging environments, and it had a large global community of developers and implementers.

### Strategy for Developing OpenMRS-Ebola

Our strategy for building the OpenMRS-Ebola EHR had four main components: (1) using Agile software methodology; (2) recruiting team members with diverse skills and experience; (3) iterative design based on usability, speed, and clinical needs; and (4) regular communication and feedback between the operations and development teams.

First, we used Agile software development approaches, which emphasize verbal communication, delivering working software early, and responding to changing requirements, to build the system. We designed our own Agile approach because none of the existing concrete agile frameworks such as Scrum matched the needs of this project. Specifically, the operations team lead (product owner) was deeply engaged with the development team, and we reprioritized and revised requirements daily; we did not have traditional sprints, and we performed Agile ceremonies such as retrospectives and showcases at the end of each release. We developed user stories, which we documented and tracked using a JIRA issue tracking system [20]. Some user stories were modified based on direct voice or email communications.

Two main teams worked on developing and deploying OpenMRS-Ebola. The operations team, stationed primarily at the ETC, comprised health information and clinical SCI personnel from the United States, the United Kingdom, and Sierra Leone. This team’s main role was to initiate the project, to help guide development based on ground-level needs, and to deploy OpenMRS-Ebola. The operations team lead acted as the product owner for this project. The development team comprised employees from ThoughtWorks (a global technology consultancy and software engineering firm), as well as OpenMRS volunteers and leadership members. The ThoughtWorks team, initially colocated in Uganda, expanded to include 10 staff members around the world. The development team had a range of competencies on developing EHR systems, from programming and project management to medical informatics and UI design.

A core component of this work involved constant communication within and among these teams. We started full-time software development with a 3-day project inception in Uganda with the development team and the lead of the operations team (who dialed in from Sierra Leone). During the project, the operations team lead and the entire development team had extensive email discussions and daily calls to share brief status updates, which also functioned as daily meetings for the development team. These were followed by a longer daily conversation between the operations team lead and key members of the development team (business analysts, tech lead, and medical informaticist) to (1) showcase work in progress for feedback, (2) reprioritize the next day’s work, and (3) agree on feature definitions that would satisfy user requirements with the least development time. The operations team regularly shared existing assessment forms, clinical workflow patterns, drug formulary lists, and other critical information with the development team to inform the design of new functionalities and UIs.

### Technical Process of Developing OpenMRS-Ebola

We initiated this project with requirements gathering on the ground by the operations lead and a volunteer effort for development that included analyzing terminology in the paper forms, adding necessary terminology to a shared concept dictionary, and building a proof-of-concept of the inpatient data collection form. Additionally, volunteers organized a hackathon in Brazil in early November that accelerated design work and developed a prototype tablet UI for use in the red zone [21].

During the project inception with ThoughtWorks, we developed a common understanding of the minimum viable product (MVP), intended end product, and foreseeable challenges. The MVP is the smallest work product that would provide value to end users. We focused on deploying the MVP as quickly as possible before moving on to develop other features. We regularly updated the list of features for the MVP and other phases based on changing needs on the ground. Ultimately, OpenMRS-Ebola had three development phases.

We followed a continuous deployment approach. We configured a continuous integration (CI) server to build and test all our code as soon as it was merged, and we used feature toggles to allow partially completed work to be integrated into the master codebase and tested by CI. We automatically deployed all successful builds to an Internet-hosted server for quality assurance and user acceptance testing. We released software updates over the Internet to training and production servers at the ETC as appropriate and as often as daily.

We built OpenMRS-Ebola on top of the OpenMRS Reference Application (version 2.1), an extensible UI with preconfigured functionality atop the OpenMRS platform [22]. We used Java and Groovy to build screens for desktop usage and AngularJS to build screens for tablet usage. We used an international, multilingual, clinical interface terminology developed for low-resource settings produced by the Columbia International eHealth Laboratory (CIEL; [23]) as the concept dictionary behind our EHR. We requested new terminology on a regular basis and incorporated new CIEL releases as often as daily during early development stages. All OpenMRS-Ebola code we wrote is free and fully open source under Mozilla Public License version 2.0 [24].

To increase development speed, we built the software as a browser-based Web application requiring constant WLAN access to the server. The operations team confirmed the suitability of this approach because of reliable power (through generators and UPS devices) and strong wireless signal (tested by walking through patients wards before the ETC opened).

Initial usability testing by the development team was carried out wearing dishwashing gloves and tinted goggles to simulate the ergonomic challenges of using a tablet in the ETC red zone.

### Design Decisions

Key design decisions focused on addressing usability, workflow, and communication problems in ETCs. In the green zone, the UI was designed to present detailed information because clinicians can review patient records in the green zone without wearing PPE. In the red zone, the UI was optimized for readability, speed, and ease of use by users wearing PPE. We designed the tablet UI for portrait mode because users found it easier to hold the tablet vertical when using one hand. The tablet functions were based on the initial workflows and forms provided by the clinical team. These functions were optimized for usability and speed so that ordering and administration of medication and intravenous (IV) fluids, as well as entry of patient status, vital signs, and symptoms, could be completed during the short bedside rounds. This led to several design decisions, including high-contrast color schemes, large buttons and text fonts, limiting the amount of information on each screen, building layouts for maximum clarity and rapid entry, limiting the use of complex numeric and text entry, and shortcuts for rapid entry of common items. At various points during development, we obtained feedback from users regarding readability and ease of use and adjusted our designs accordingly.

### Hardware Selection

We chose the Sony Xperia Z2 10.1-inch tablet for our EHR because it was waterproof and could be disinfected with chlorine. We charged the tablet using the Sony magnetic charging dock DK39. Clinicians who preferred a stylus instead of their gloved finger used the Boxwave EverTouch Capacitive Stylus with fiber mesh tip that plugged into the tablet headphone jack.

### Implementation

OpenMRS-Ebola was deployed in phases at the ETC, with the most essential functions deployed in the MVP (phase 1). Key implementation components involved field testing at the ETC before deployment, training sessions for staff on how to use the hardware and OpenMRS-Ebola software, installing tablets in the red zone patient wards and laptops in the green zone areas, installing OpenMRS-Ebola onto the ETC server, and obtaining user feedback to improve the software.

### Evaluation

In February 2015, we asked clinicians to complete a predeployment structured questionnaire about their experiences with the existing paper-based records system and thoughts about an EHR. However, a postdeployment questionnaire was not conducted because of staff departures after the ETC closed. We also obtained informal feedback from users during development, field testing, and deployment.

Patient data were entered in both OpenMRS-Ebola and the existing paper-based record system for more than a month after the rollout of phase 1 while we made changes based on user feedback. We compared the patient registration and drug-ordering data entered for each patient in both systems to identify differences and to evaluate how the EHR was functioning compared with the standard paper-based system. Although phase 2 was deployed in mid-March, we were unable to do a similar comparison because it was not fully used due to the ETC closing.

## Results

### Software Product

The final OpenMRS-Ebola EHR includes a browser-based desktop or laptop application for the green zone and a browser-based tablet application for the red zone, each with different UIs but accessing the same data and software infrastructure. Both applications have separate but overlapping modules and functions. The desktop application comprises six key modules for managing patient tracking, entry of some clinical information, and viewing detailed patient summaries (Table 1). The tablet application has five key modules, primarily focused on the following common red zone tasks: drug and IV fluid ordering and administration, entry of patient vital signs and symptoms, and viewing limited patient summaries (Table 1).

**Figure 1:**
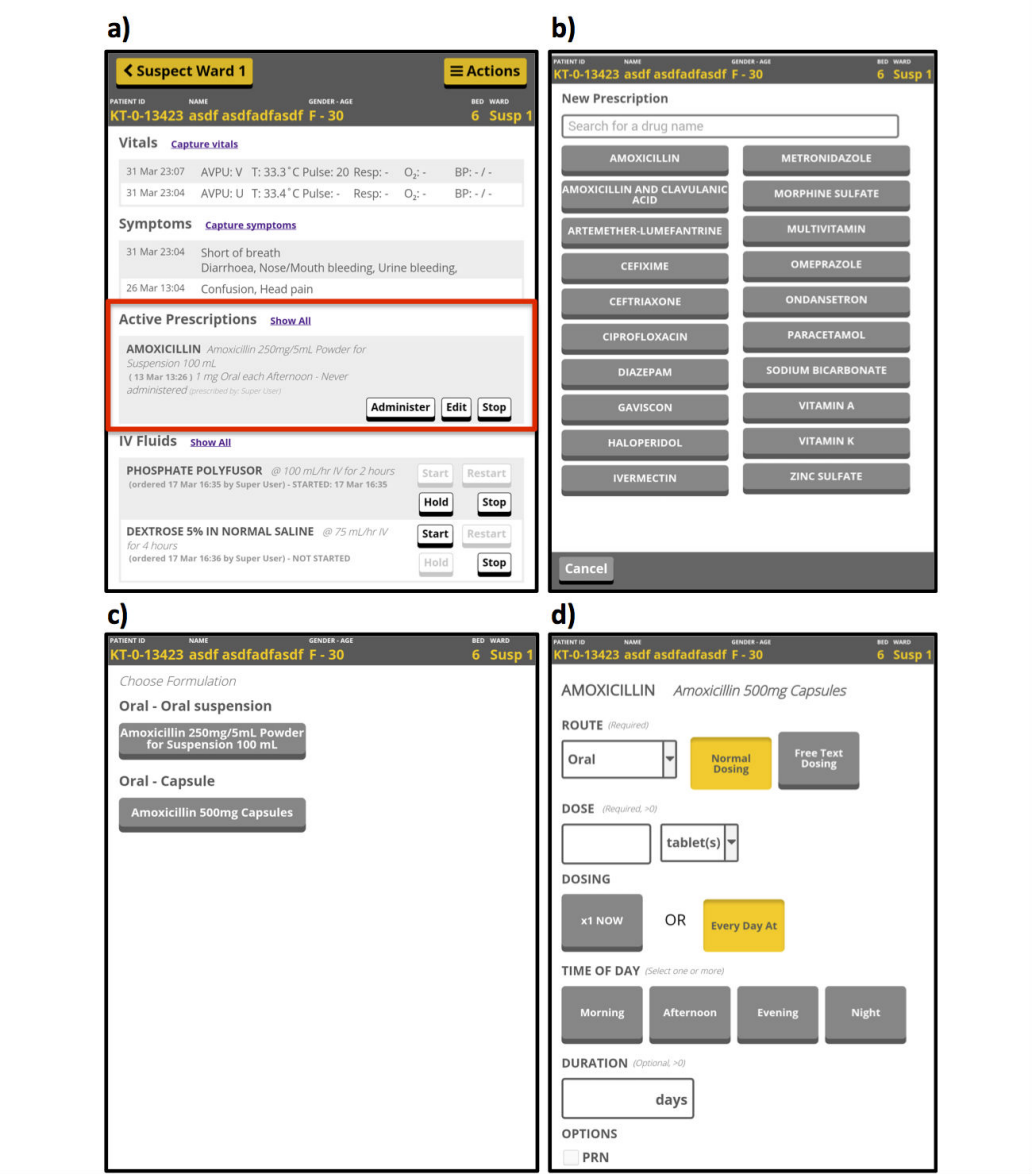
Medication ordering in the tablet application, designed for rapid entry while wearing personal protective equipment (PPE). The patient summary page (a) shows active prescriptions, including buttons to record medication administration and stoppage. Selecting “order medication” from the Actions menu brings up the drug choice page (b), including rapid selection of the 20 most common drugs (accounting for over 90% of orders) and an update-as-you-type search control to access other drugs by name. The next page (c) offers available form and strength options of the selected drug. Dosing instructions are entered (d) based on the Ebola treatment center (ETC) workflow, which had standard rounds per day.

**Figure 2:**
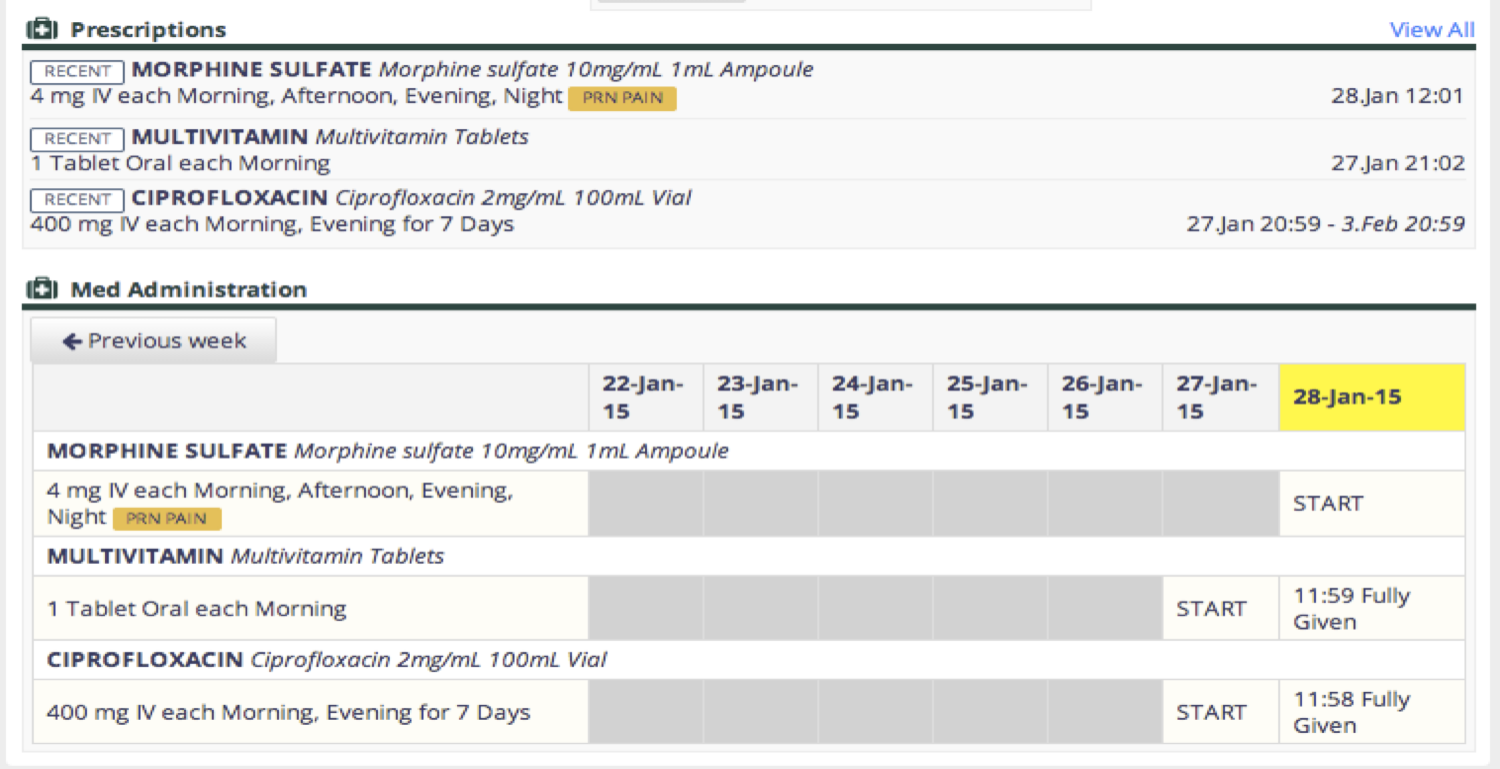
Desktop-based prescription and medication administration charts.

A detailed example of a module, tablet-based drug ordering and monitoring, is shown in Figure 1. Additional desktop-based functions for this module include complete drug charting with medication administration time stamps and a list of recently edited prescriptions for pharmacy review (Figure 2). A similar module was designed for IV fluid ordering and monitoring (see Multimedia Appendices 1 and 2).

**Table 1:** Modules and functionalities of the OpenMRS-Ebola electronic health record (EHR) desktop and tablet applications.

Modules and functions		Description	Application type^a^	Rollout phase^b^
**Patient tracking**				
Registration		Date, name, demographics, contact information, ID # allocation, quick assessment^c^	Desktop	1
Bed allocation		Selection of ward and bed #	Desktop	1
Discharge		Date and patient outcome	Desktop	1
**Clinical input**				
Medications		Ordering, medication administration, and discontinuation	Tablet	1
IV^d^ fluids		Ordering and administration (with start, hold, restart, and stop functions)	Tablet	2
Patient vital signs		Key vitals, including temperature, pulse, blood pressure, and consciousness level	Tablet	2
Symptoms		Key symptoms, patient status, and observations	Tablet	2
Laboratory tests		Ebola and malaria results by date	Desktop	3
Clinician notes		Time-stamped free text note entry	Desktop	3
**Clinical output**				
Detailed patient summaries		Full patient details: patient demographics and bed location, vitals, symptoms, medications, full medication administration chart, IV fluids, labs, and clinician notes	Desktop	2
Abridged patient summaries		Patient demographics and bed location, recent vitals and symptoms, active prescriptions, and IV fluids (expandable to full history)	Tablet	2
**Additional functionalities**				
Active patients		List of active patients by ward with bed #s	Desktop	1
Data editing		Ability to retrospectively edit data as needed	Desktop	1, 2
Data export		Export data from modules to CSV^e^ files	Desktop	3

^a^Functionality designed for the tablet application is responsive to different screen layouts and can also be used on the desktop or laptop.

^b^Rollout phases: Phase 1 (deployed in mid-February), phase 2 (deployed in mid-March), and phase 3 (development completed in late March but not deployed because of Ebola treatment center [ETC] closing).

^c^Type of patient (confirmed or suspect), stage of illness.

^d^IV: intravenous.

^e^CSV: comma separated values.

**Figure 3:**
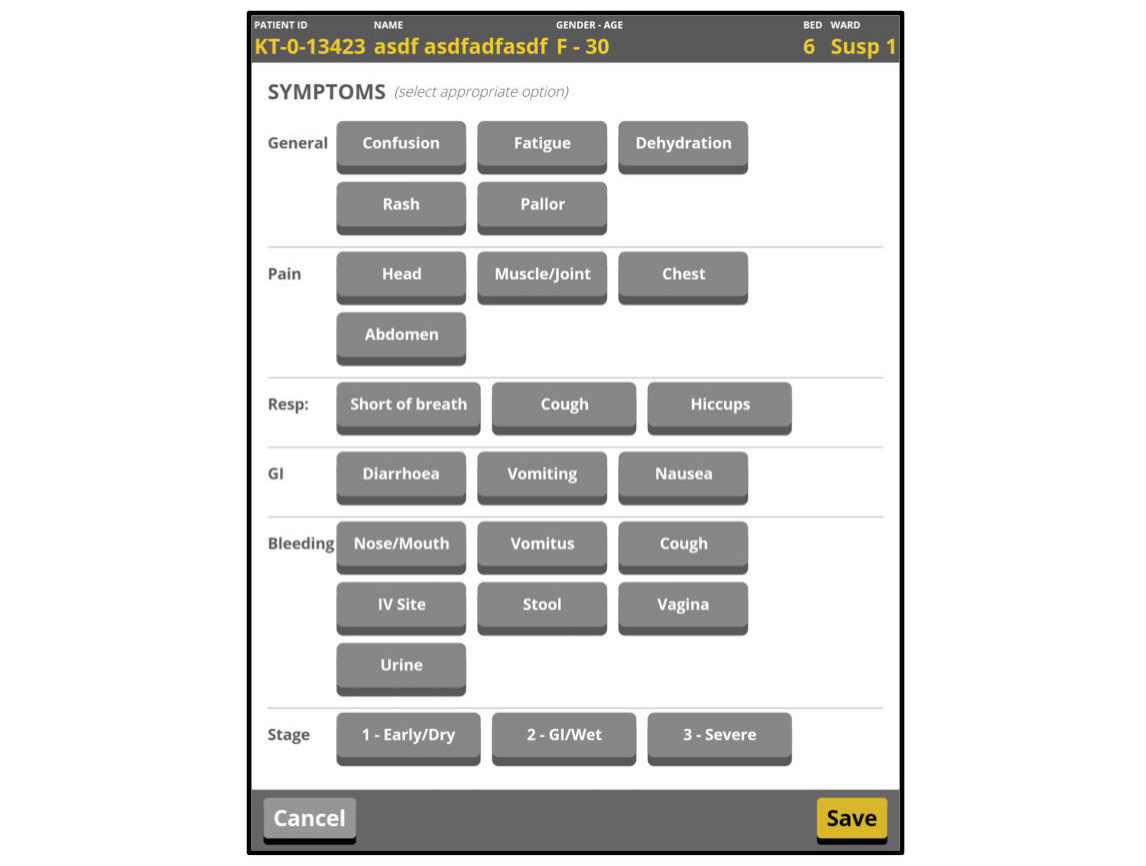
Symptom assessment module in the tablet application.

The patient symptom assessment presented particular challenges to make it fast, usable, and meaningful on the tablet. We discussed several tablet application designs with the operations team before reaching a satisfactory single-page button-only form (Figure 3).

**Figure 4:**
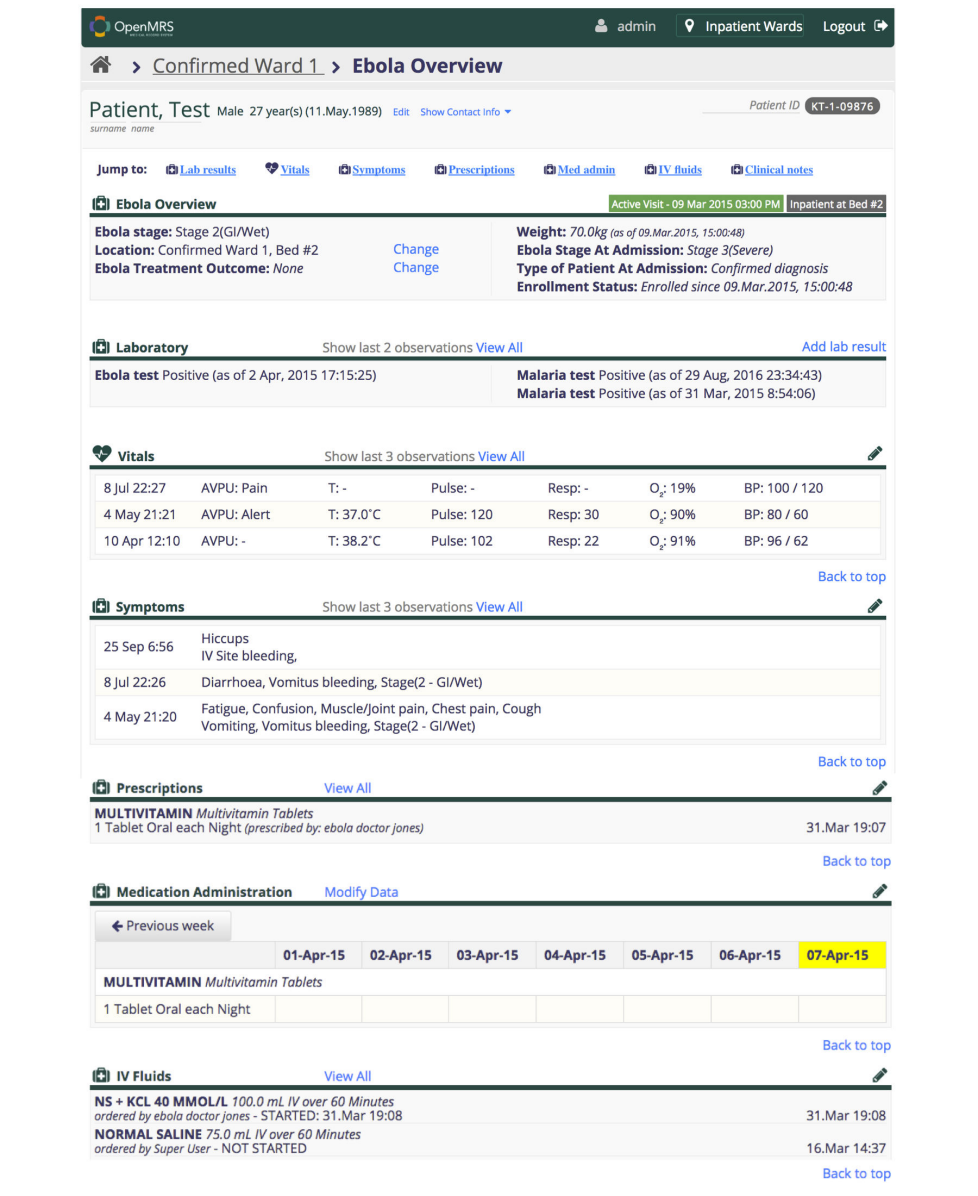
Full desktop summary.

An example of the desktop patient summary is shown in Figure 4. This screen includes recent information for all recorded clinical data and options to view the full clinical history during the ETC stay.

Screenshots of the full desktop and tablet Ebola EHR applications, with all modules, are included in Multimedia Appendices 1 and 2.

### Cost

SCI paid approximately US $50,000 for OpenMRS-Ebola, with about US $38,000 to ThoughtWorks for software development and US $12,000 for hardware. However, the true cost of developing and deploying OpenMRS-Ebola was approximately US $260,000, the majority of which was donated by ThoughtWorks through staff time. The full costs (excluding proof-of-concept and predevelopment volunteer work) were estimated to be US $187,000 for ThoughtWorks staff (based on reported hours and social impact project rates); US $6,000 in ThoughtWorks equipment and travel; US $12,000 by SCI for tablets, charging docks, and styluses; US $50,000 in donated time by 3 key volunteers; and about US $5000 for dual-use hardware already at the ETC (ie, server, 2 laptops, wireless routers, and UPS devices). The cost of redeploying the system with adaptions in a new emergency would be considerably less with reuse of the workflow, UI designs, and code.

### System Deployment and Usage

Volunteer programmers began work on OpenMRS-Ebola in November 2014. ThoughtWorks began full-time development in early December 2014. Phase 1 was completed and deployed in mid-February 2015, with user testing and feedback starting in January while the modules were being finalized. Phase 2 was completed and released in mid-March, and phase 3 development was completed in late March. Phase 3 was not deployed because the Kerry Town ETC closed on short notice at the end of March 2015 after a dramatic decrease in new Ebola cases. Therefore, the phase 2 and 3 modules saw little to no use on actual ETC patients.

From January to March 2015, nearly 100 clinicians were trained to use the system. In total, 112 of 456 Kerry Town ETC patients were registered in OpenMRS-Ebola. All but 2 of those not entered into OpenMRS-Ebola had completed their ETC stay before the system was deployed. The 2 remaining patients were discharged within hours of being admitted. For drug ordering, 569 prescription orders were placed using the system, and 971 medication administrations were recorded.

### Evaluation

Sixteen clinicians completed the predeployment questionnaire, including staff from Sierra Leone (n=5), Cuba (n=6), and other countries (n=5). They included the full range of clinical staff, from community health assistants and officers to nurses and doctors. The questionnaires were completed in February 2015, 3 months after the ETC opened. When asked whether they agreed or disagreed that an electronic patient record system could improve patient records at the ETC, 7 strongly agreed, 8 agreed, and 1 was neutral. Some reasons they stated for wanting an EHR over the paper system included likelihood of higher quality data collection in the red zone, not having to rely on memory for transferring data to the green zone, real-time data collection and review, records not being damaged or missing, and legibility. Their concerns about a potential EHR included adoption by users without previous tablet or EHR experience, difficulty if training is inadequate, power outages, equipment breaking, having parallel systems if the EHR could not fully replace the paper system, and cost.

Although we were unable to complete postdeployment user questionnaires, we obtained informal feedback about the EHR during the phase 1 and 2 field testing and deployments. Positive comments included that OpenMRS-Ebola was easy, fast, and intuitive to use; the UI worked well with red zone visibility or dexterity problems; the EHR seemed useful for both red and green zone entry and review; and that the EHR would be favored over the paper system if there was more training and if network connectivity was good. Concerns included needing more training for users unfamiliar with such systems, potential delays in fixing bugs, and having to use both paper and electronic records during the phased rollout.

When comparing the paper and EHR records for the 112 patients registered in OpenMRS-Ebola, ID numbers, age, and sex were incorrectly recorded in the EHR 4, 2, and 2 times, respectively. In comparison, a basic pre-EHR database created for use with the paper records initially had 7, 5, and 3 errors when recording ID numbers, age, and sex, respectively, for these same patients. For the prescription entry, 97.2% (553/569) of the prescriptions in the EHR system correctly matched the paper records. Of the remaining 16 EHR prescriptions, 4 were missing from the paper drug charts but recorded elsewhere in the paper notes, 6 were missing from the paper charts altogether, and 6 were for a patient with missing paper records. A total of 77 prescriptions were recorded in the paper system but not in the EHR. Of these, 67 had specific identifiable reasons: 30 were prescriptions given as needed (pro re nata or PRN) without pharmacy prescription, 24 were illegible or ambiguous, and 13 were because of drugs not listed in the EHR during the first weeks of deployment. The latter were reported as missing and subsequently fixed in the EHR system.

The time taken to perform this evaluation was also informative. The majority of time spent on analysis for this evaluation was because of the time-consuming nature of working with paper records. The code to analyze the EHR data took minutes to write but going through the paper records took days. Additionally, legibility was a major issue for some of the paper records, and missing or damaged pages were a less common but still important problem.

## Discussion

### Principal Findings

We rapidly developed an open-source Ebola EHR system that was deployed at Save the Children’s Kerry Town ETC during the West African Ebola outbreak. OpenMRS-Ebola was designed specifically to address the many challenges in recording patient data within ETCs that arise from severe infection control measures. This EHR supports registration, bed allocation, and discharge of patients; recording of vital signs and symptoms; medication and IV fluid ordering, administration, and monitoring; laboratory results; clinician notes; and data export for analysis. It displays relevant patient information to clinicians in both the infectious and noninfectious zones. To our knowledge, this system is the most comprehensive clinical EHR built for Ebola and is able to function as a stand-alone medical record system in an ETC.

The evaluation suggested that OpenMRS-Ebola worked well in the context of our rollout. There were two main sources of error, with both being ones that would be expected during a rollout. First, some errors were discovered during implementation, such as an initially incomplete drug list that resulted in a few missing prescriptions. Second, during the initial implementation of the EHR, clinicians used it in parallel with the existing paper system. This meant that it was less than fully integrated into the clinical workflow, and some users may have taken the system less seriously than if the parallel paper system was not required. One example of this is the 30 missing PRN drugs (given in the red zone without a pharmacy prescription), for which the medication administration recording workflow differed between the paper and EHR systems. The OpenMRS-Ebola records had fewer age or sex or ID errors than the pre-EHR (ie, single-entered) database for the paper records. Although those initial database errors were fixed through careful checking of the paper records, this is a time-consuming task. One explanation could be that clinical staff familiar with the patients entered data into the EHR, whereas the database for paper records was completed by data entry clerks who were retrospectively copying from handwritten, possibly illegible, paper charts. This analysis also demonstrates the value of the EHR in terms of how much quicker it is to enter, analyze, report, and evaluate data compared with a paper-based system.

### Strengths and Weaknesses of Approach

Although we successfully built the full OpenMRS-Ebola EHR system and implemented its first phase, this system’s usefulness was limited because it was deployed around the time when the number of new Ebola cases began to decline in Sierra Leone. The Kerry Town ETC closed soon afterward, just as we were deploying phase 2. This is a major drawback of developing a system during the outbreak. At the time we began building the system, the number of Ebola cases was increasing exponentially, and many believed that the outbreak would last a long time [25]. We therefore had to plan for a wide range of options, including the likely possibility that the Kerry Town ETC would be open for many months. We also hoped to share our EHR with other ETCs because they were all struggling with the same data communication issues between red and green zones and had similar workflows, triaging, and patient care. We believe that OpenMRS-Ebola would have shown greater value as the epidemic continued and may have had wider adoption. Fortunately, the epidemic forecasts when we were developing OpenMRS-Ebola in autumn 2014 were more pessimistic [25,26] than the reality [1], and the epidemic wound down more quickly than most expected. The design experience, added functionalities, and lessons learned from this project should be applicable to future health emergencies, making earlier deployment of an EHR plausible.

We tried to minimize the inherent problem of software development during a health emergency, namely that software requires planning but rapidly evolving situations mean dynamic needs. We first gathered information on the ground to determine how best an Ebola EHR could be designed to address the challenges encountered with the paper-based records system. We started developing OpenMRS-Ebola as the Kerry Town ETC opened, and we recognized that our ideas and designs needed to continuously evolve with changing needs and processes on the ground. To do this efficiently, we selected highly flexible methods, including a modular software platform, Agile software methodology, daily communication between teams, frequent feedback from users on site, regular reevaluation of priorities, and a phased implementation.

We benefited from working with a diverse group, from clinicians and epidemiologists to programmers and UI designers. Our team was located across six continents, which could have produced collaboration problems but instead was beneficial both for perspective and the ability to effectively work around the clock. Several individuals, including the project leads on the operations and development teams, remained on the project from start to finish. This was essential for developing a usable product during rapidly changing conditions but also unusual because of the typical high staff turnover of field staff (eg, operations lead) during such responses.

A key challenge for this work was the dynamic situation on the ground. The clinical workflow at the ETC shifted based on changing clinical protocols, unfamiliarity with treating large numbers of Ebola patients, and frequent turnover of the clinical director and short-term medical teams. For example, drug ordering and monitoring were done at the patient's bedside in the red zone when the ETC opened. Correctly communicating these orders to the green zone was very difficult, especially given the large distances between the red and green zones at this ETC. Since this could have serious safety implications for patients, drug ordering and monitoring were deemed to be a top priority for the EHR and were included in the MVP. However, about 2 months later, the clinical staff changed their workflow to order drugs from the green zone instead, which was nonideal but safer for patients given the lack of instant and accurate communication between zones. The tablet-based drug-ordering module was time-consuming to build but already largely developed at this point. If the software had been ready earlier, the clinicians likely would not have changed their drug ordering workflow, and prescriptions could have been done more safely from the red zone.

One implementation challenge was the limited time to introduce and train staff on the system. We conducted a large showcase, where the development team dialed in to present the product, for all available users before OpenMRS-Ebola was deployed. However, we found that smaller group launches worked better in our setting and should be the norm in health emergencies. For training, we held sessions where clinical staff were able to use and learn the OpenMRS-Ebola interface. We combined our EHR training with more general training on common programs such as Microsoft Word and Excel. This helped increase staff engagement and computing familiarity for those new to such technology. More consistent training and retraining when necessary are critical for the success of new technologies in such situations. This is particularly difficult during an emergency response where hundreds of clinicians, sometimes rotating every few weeks, may provide patient care. The need to adequately train the user must also be balanced with the need to urgently deploy the system. One important consideration is whether all clinical staff should be trained to use the system or if a select set of superusers can mediate all interactions with it. This decision depends at least, in part, on the clinical workflow and skill levels of team members.

Regular communication between the operations team and system users is an important component of user support and buy-in. We had an operations team member speak at morning clinical meetings and discuss overnight software updates with the clinical lead. We also consulted clinicians on how to redesign the clinician station to fit the EHR system and regularly had an operations team member at the station to answer questions about the system as clinicians entered and exited the red zone. We found this difficult to maintain, especially with staff turnover, but it is essential for a smooth and rapid rollout.

We also faced staffing challenges on the ground, including being able to correctly time the deployment of an experienced team member to lead the EHR implementation, given the difficulty in determining when the MVP would be released. An effective champion on the ground is necessary for a successful implementation, especially during a health emergency where there are few chances for a do-over. Having an organized and properly staffed operations team, both in terms of personnel and skill levels, is critical for a successful rollout. This is especially true during an emergency when there are many demands for health information, including daily reporting to external actors. Deploying our EHR required some effort, at minimum, from an operations lead, a health information manager, 3 to 4 additional health information staff, a key clinician, 3 to 4 additional go-to clinicians, and information technology (IT) support (both on the ground and remote). Without knowledgeable IT staff on the operations side, even a complete EHR may not be deployable on the ground. For our deployment, the SCI IT staff needed to urgently solve network access control and security issues and build a virtual Linux machine on a Windows server. Such requests are not typical IT needs for most emergency responses, so it is essential that proper and accessible IT support is secured before deploying an EHR in such situations. Ideally, local staff can be trained to fill several of the operations positions needed for implementation, which is useful both for the project and overall capacity building. Partly because of the experience received with deploying OpenMRS-Ebola, at least one local health information staff member obtained subsequent employment in EHR-related work at a local hospital after the epidemic.

Finally, a common issue for EHRs—especially in low-resource settings and emergency situations—is lack of evaluation data on performance, usage, and potential evidence of benefits to patient care and health facility management [27,28]. During emergencies, making a case for research or carrying out reliable studies is even harder. Yet, without such data, developing and deploying effective systems will remain an uncertain process and may be hard to justify. We were able to do a partial evaluation because we had a predeployment questionnaire and entered data in both the electronic and paper record systems for about a month as we rolled out phase 1 of the EHR. However, we were unable to perform a more complete evaluation because the ETC closed earlier than expected as the epidemic began winding down. Given the volatile nature of emergency responses, preplanning evaluations and having ideas in place on how to complete the evaluation in changing circumstances is important.

### Comparison With Other Work

We conducted a literature review in March 2017 for publications regarding eHealth information systems for supporting the management of Ebola. We found 17 papers after searching PubMed for “Ebola and (“EMR” OR “EHR” OR “Medical Record” OR “mHealth” OR “eHealth” OR “mobile health”).” After reviewing titles and abstracts, we assessed 4 as relevant. These were supplemented with 3 papers recommended by colleagues from the gray literature and from an April 2015 meeting of team leaders from several projects hosted by the International Rescue Committee (IRC). To date, there are only a few publications that describe the development and deployment of EHR systems during the Ebola outbreak and only one that describes an EHR for use in ETCs [5].

No EHRs suitable for ETCs existed when we began developing OpenMRS-Ebola. Around the same time as us, a few other ETC EHR projects were initiated. Médecins Sans Frontières (MSF) partnered with Google Inc to develop and deploy Project Buendia, a basic tablet-based EHR [5]. They also developed robust hardware, including a waterproof tablet enclosure with charging capabilities and a miniature server with built-in low-power backup that allowed offline use with later synchronization of data. VecnaCares partnered with IRC to build an EHR with CliniPAK [29], and Project ELEOS was launched by MSF Belgium to deploy a simple data collection protocol on a personal digital assistant (PDA; [30]).

These other EHR systems, however, recorded a subset of the data that OpenMRS-Ebola collects, and not all were implemented during the outbreak. In particular, they lacked several important functions such as drug and IV fluid ordering, administration, and monitoring. Such data are complex to collect accurately, and errors can put patients at serious risk. Drug order entry systems are considered critical parts of successful EHRs but are rare in low-resource settings.

OpenMRS-Ebola and Project Buendia shared important components. Project Buendia was also built using the OpenMRS platform that was linked through OpenMRS application programming interfaces (APIs) to an Android application. Some clinical vocabulary—a subset of the CIEL dictionary focused on Ebola—was shared with Project Buendia to help speed up development. Project Buendia started in September 2014 and deployed in March 2015, at a cost of about US $1.9 million [5]. Although there was some contact between the OpenMRS-Ebola and Project Buendia teams, the projects diverged early on because of different requirements. In particular, unlike MSF sites, the Kerry Town ETC had unusually reliable power and infrastructure as part of the United Kingdom government's Ebola response. Thus, we were able to save time by developing a browser-based application, whereas Project Buendia needed an application that allowed offline use.

Overall, we believe that our system is the most comprehensive adaptable clinical EHR software developed to date for a health emergency in a low-resource setting. The potential for this system is strengthened by the wealth of software designs and code modules already deployed worldwide by projects using OpenMRS and a growing evidence base of the impact of OpenMRS use on care processes [31,32].

### Speeding Up Use of Health Software During Emergencies

**Figure 5:**
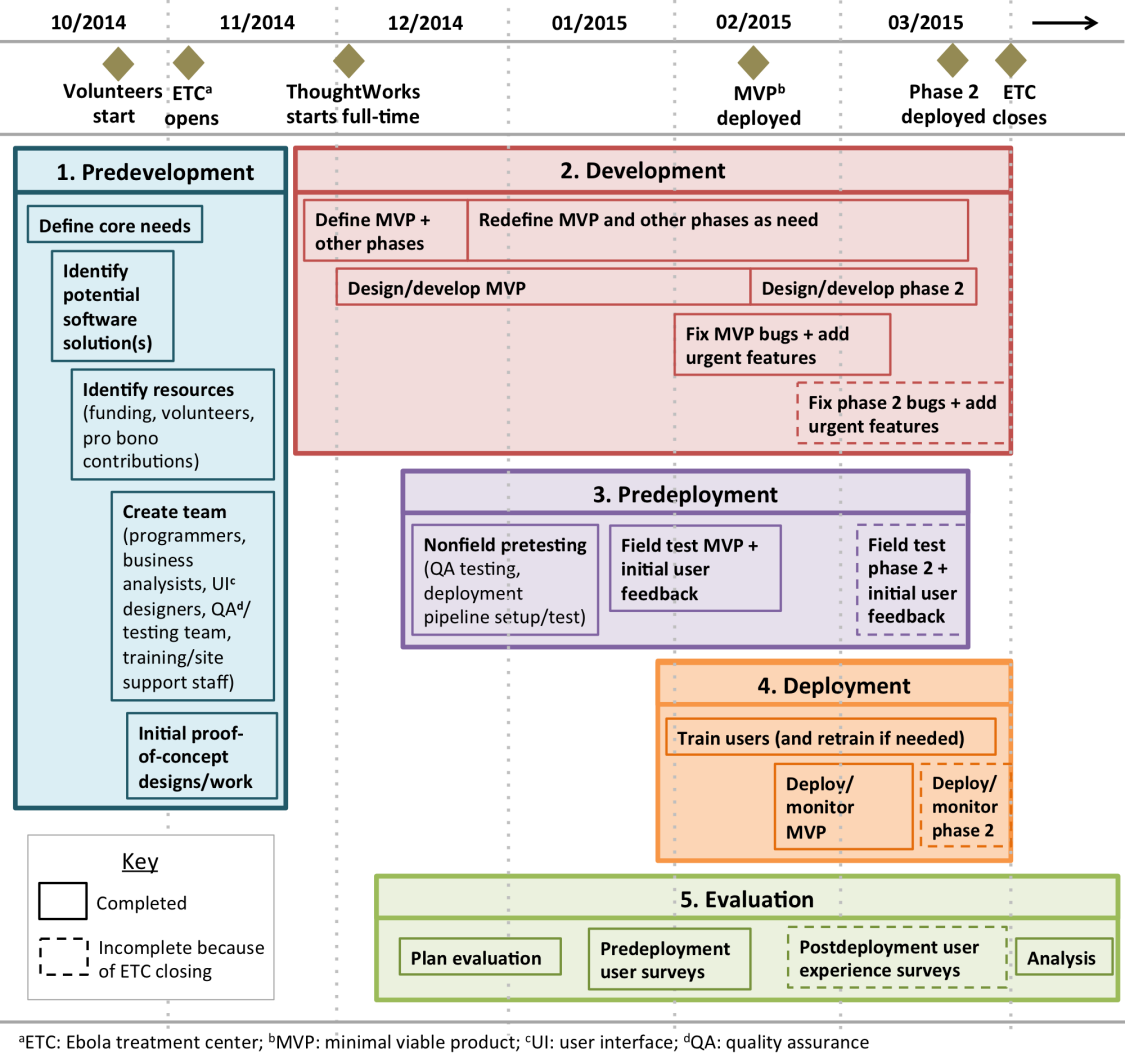
Stages in development and deployment of an electronic health record (EHR) during a health emergency, using OpenMRS-Ebola as an example (note: phase 3 development is not included).

A key contribution from this project has been to advance our understanding of the process required to develop and deploy EHRs for emergencies in low-resource settings, as well as defining the factors slowing this process and how to address them. Figure 5 shows the stages that we recommend for deploying an EHR in such a setting, using the example of OpenMRS-Ebola, and potential areas for improvement.

The biggest delays for deploying an EHR at the Kerry Town ETC occurred for four main reasons. First, the largest delay occurred because no suitable off-the-shelf EHR existed that could be immediately adapted and put into use at the ETC. Second, the lack of a suitable generic tablet interface for OpenMRS meant that the platform could not be immediately adapted for the most critical need: use in the red zone. Third, although volunteers made major early contributions, it took us a month to realize we needed full-time staff (eg, programmers, UI designers, and business analysts) to complete the EHR as quickly as possible and to contract that full-time development team. Finally, we faced the standard challenge of doing requirements gathering, field testing, and implementation under emergency conditions. This included a late start to field testing and user training because of a long delay in receiving the Sony tablets and other key hardware.

On the basis of our experience with OpenMRS-Ebola, we have listed a set of recommendations for rapidly building, deploying, and evaluating an EHR during a health emergency in Table 2. Even before the health emergency, however, work should be done to design and develop components that are necessary but lacking based on experiences from prior emergencies and anticipated functional needs.

**Table 2:** Recommendations for rapidly building, deploying, and evaluating an electronic health record (EHR) during a health emergency.

Process stage	Recommendations
Predevelopment	Hire full-time professional staff to complete the product (ie, do not rely purely on volunteer efforts)
	Select a product owner who is a key stakeholder, deeply knowledgeable of the ground-level needs, and will remain engaged throughout the project
	Ensure that development team is proficient in Agile software approaches
	Set up mechanisms for regular (daily) communication
	Test and select hardware early to ensure (1) suitability based on needs (eg, waterproof, low power, and long battery life) and (2) that software designs are compatible with hardware
	Conduct review of working environment, clinical situation, and needed functionality with health workers
Development	Define the MVP^a^ based on both ground-level priorities and time to develop features
	Communicate with ground-level team at least daily, if possible, including demonstrations and review of work in progress
	Conduct operational assessments of hardware and infrastructure needs
	Reprioritize MVP and other phases regularly based on ground-level feedback
Predeployment	Create communication mechanisms for user feedback to reach development team regularly and set up test and training servers to support this
	Ensure that operations team has appropriate staffing and skills required for EHR^b^ rollout and troubleshooting
	Prepare training materials in advance and have plans for training and refresher training
	Make sure all hardware is ordered well in advance of user testing and training
	Determine strategy for selecting and training users (eg, all users vs superusers)
	Set up deployment pipeline to load and update software on production server
	Create contingency plans for anticipated problems (eg, locks for hardware, backup paper data collection, and backup power supplies)
Deployment	Ensure that deployment lead is proactive and can create and maintain buy-in from staff
	Conduct regular trainings with user-friendly material (eg, videos and annotated examples) and refresher trainings when needed
	Confirm that communication and feedback channels with development team are functional
Evaluation	Plan evaluation (including templates for pre- and postdeployment user surveys) during early development
	Keep records of informal feedback throughout the process
	Plan for contingencies (eg, obtaining consent and contact information for a Web-based follow-up user survey if emergency ends earlier than anticipated)

^a^MVP: minimal viable product.

^b^EHR: electronic health record.

### Future Work

Ideally, the best time to prepare for an emergency response is before the emergency. In this outbreak, the key reason no organization was able to fully implement an EHR before ETCs began closing was because we had to do real software development during the outbreak. Learning from this and previous emergency responses can help identify common themes and modular configurable software that can be made at least partially ready in advance. Although EHR advances by us and others during this outbreak have helped fill many of the gaps we encountered at the start of this work, more development is still needed to extract and extend this work into a comprehensive EHR system suitable for health emergencies. Additional features would include laboratory test ordering, clinical decision support, pharmacy dispensing, integrated data entry quality checks, and customizable automated reports, as well as linkages to external laboratory services, community-level care, and surveillance. Coupling this with hardware advances, such as those started by Project Buendia, can result in a complete product that is ready to go out of the box after minor adaptations. Most importantly, the time to work on further advancing these software and hardware features is now, instead of waiting until the next health emergency hits.

More generally, responding to outbreaks such as this one requires effective and rapidly deployable strategies across a range of health activities, including case detection, triage, contact tracing, public education, and treatment of infected patients. This requires a set of robust technologies that are semantically interoperable and easily adaptable to support high-quality data collection during emergencies. Several open-source applications already exist that fill different niches in the health system, have been implemented at the national scale, and support interoperability and secure data sharing. These include DHIS 2 at district- and national-level for aggregate data; OpenMRS, Baobab Health (Malawi), iSante (Haiti), and others for health facilities; and several mobile technology platforms in the community such as CommCare and RapidPro. Some of these were deployed in this outbreak and projects such as mHero, a communication tool between health workers and health ministries, combined more than one system [33-36]. However, these systems were largely deployed on an ad-hoc basis and not as part of a network of integrated health information technologies that could efficiently fill complementary data needs. To get to that point, these systems need to be reliably interoperable, easily adaptable, user friendly, and well tested with predesigned and accessible training materials.

Without better coordination and communication—both between and within developer and implementer teams—technology will not be efficiently implemented during an emergency, even if the software is fully ready. A detailed registry of suitable software, ideally managed by the World Health Organization or another international organization, is needed. This would provide a centralized location for governments and emergency response organizations to find and compare IT options, including interoperable ones, at the start of a health emergency instead of doing ad-hoc searches or relying on word-of-mouth contacts. ICT Africa tried but failed to obtain funding for such a registry in 2016 [37]. If we want to be better prepared for future emergencies, we need to fund and maintain such efforts. Similarly, a centralized and easy-to-use site to communicate with other responding organizations could improve coordination during an emergency and promote shared solutions. For example, we connected with other organizations developing EHRs through personal contacts, but a platform to share our work, ideas, and experiences would likely have sped up development and implementation. Such tools may be especially useful for rapid emergency deployment teams, which have increased substantially since the Ebola epidemic. Finally, it is essential that organizations be open and collaborative about their efforts during an emergency, as this can speed up development and implementation. The free and open-source software approach is a powerful framework for collaboration, not only across nonprofits but also with a larger community of developers. At the time of their development, none of the other Ebola EHR projects were open source. As software can be shared at no cost, we think that such cooperation among nonprofits with a common mission should become routine.

Ultimately, though, the best way for relevant technologies to be rapidly deployed in a health emergency is for them to already be integrated within the standard health system. There is understandable reluctance to introduce untried systems and approaches during emergencies, and new technologies come with standard challenges such as additional staff training, setup costs, and potential glitches. For nearly a decade, deploying electronic systems such as EHRs in LMICs in a scalable fashion has been possible [38]. If those systems are developed and deployed using common terminology and coding standards, they can be expanded and adapted for crises while strengthening the core codebase. OpenMRS is now implemented at a national scale in several countries and has been deployed in eastern Sierra Leone after the Ebola outbreak as a standard part of data collection at a large primary care clinic [39]. Systems such as DHIS 2 have also become increasingly popular in Sub-Saharan Africa [40]. Increasing such integration is a needed step toward EHRs becoming commonly used tools in health systems worldwide. There is also an increasing interest in the use of tablets for EHR users in LMICs, and lessons learned from this project should help the design and implementation of such systems.

Due to its fast and easy-to-use interface, OpenMRS-Ebola has potential to help with data collection for a range of other clinical needs (crisis and routine) in LMICs. Its features and adaptability, for example, make it suitable to support data collection in intensive care units and infectious environments. Similarly, the collection of vital signs is a key step in implementing early warning scores for clinical deterioration in hospitals (NEWS Scores). Systems such as OpenMRS-Ebola can help automate that process, similar to innovative systems developed for Oxford hospitals in the United Kingdom [41]. A key priority should be to expand the use of well-functioning technologies into standard health systems. Having the technology already integrated in the health system means having the benefits of an EHR during nonhealth emergency times and an easier transition to high-quality data collection during an emergency response.

### Conclusions

The OpenMRS-Ebola EHR is well suited for patient records in an ETC because it allows for instant communication between infectious and noninfectious zones over a local wireless network, access to full clinical histories in both zones, and has a fast, easy UI suited to this difficult environment. Careful user design on a flexible platform can rapidly yield EHRs that are suitable for health emergencies. We were relatively successful in rapid development and deployment, but better preparation could likely have reduced time to implementation of the full system by approximately half (about 2 months). OpenMRS-Ebola can be adapted for future emergencies and is interoperable with other eHealth systems. To make a real impact, however, it must be part of a well-designed and tested set of interoperable electronic systems ready for deployment with appropriate hardware and training materials. Health information is too important a resource in emergency situations to be treated as an afterthought.

## Appendix

Multimedia Appendix 1 Complete screenshots of the desktop or laptop-based OpenMRS-Ebola application.

Multimedia Appendix 2 Complete screenshots of the tablet-based OpenMRS-Ebola application.

## Abbreviations

API: application programming interface
CIEL: Columbia international eHealth laboratory
CI: continuous integration
CSV: comma separated values
ETC: Ebola treatment center
eHealth: electronic health
EHR: electronic health record
HIV: human immunodeficiency virus
IRC: International Rescue Committee
IT: information technology
IV: intravenous
LMICs: low- and middle-income countries
mHealth: mobile health
MSF: Medècins Sans Frontiéres
MVP: minimum viable product
PPE: personal protective equipment
PRN: pro re nata
SCI: Save the Children International
TB: tuberculosis
UI: user interface
UPS: uninterruptable power supply
VSAT: very small aperture terminal
WLAN: wireless local area network
