# Supplementary material for: Development and Deployment of the OpenMRS-Ebola Electronic Health Record System for an Ebola Treatment Center in Sierra Leone
Source: J Med Internet Res. 2017 Aug 21;19(8):e294. doi: 10.2196/jmir.7881 (PMC5583502; doi:10.2196/jmir.7881)

Development and deployment of the OpenMRS-Ebola Electronic Health Record System for an Ebola Treatment Centre In Sierra Leone

Shefali Oza, Darius Jazayeri, Jonathan M. Teich, Ellen Ball, Patricia Nankubuge, Job Rwebembera, Kevin Wing, Alieu Sesay, Andrew S. Kanter, Glauber Ramos, David Walton, Rachael Cummings, Francesco Checchi, Hamish Fraser

Multimedia appendix 1:

Complete screenshots of the desktop/laptop-based OpenMRS-Ebola application

Primary use – Green (non-infectious) zone of Ebola Treatment Center

Note: All patients and data included here are fictitious

Desktop: login and dashboard

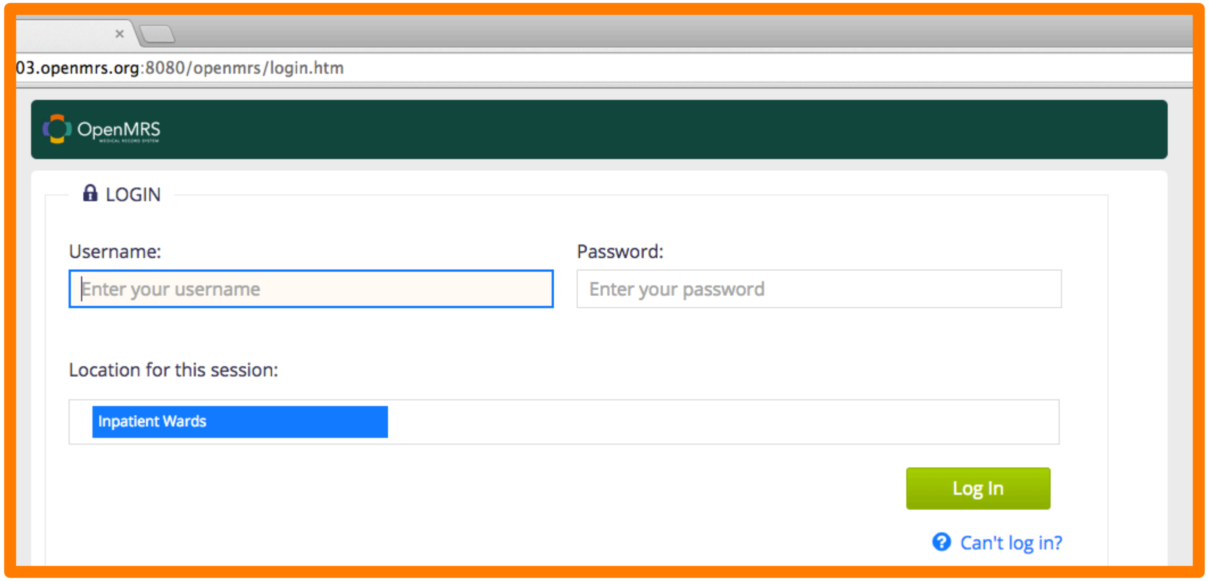

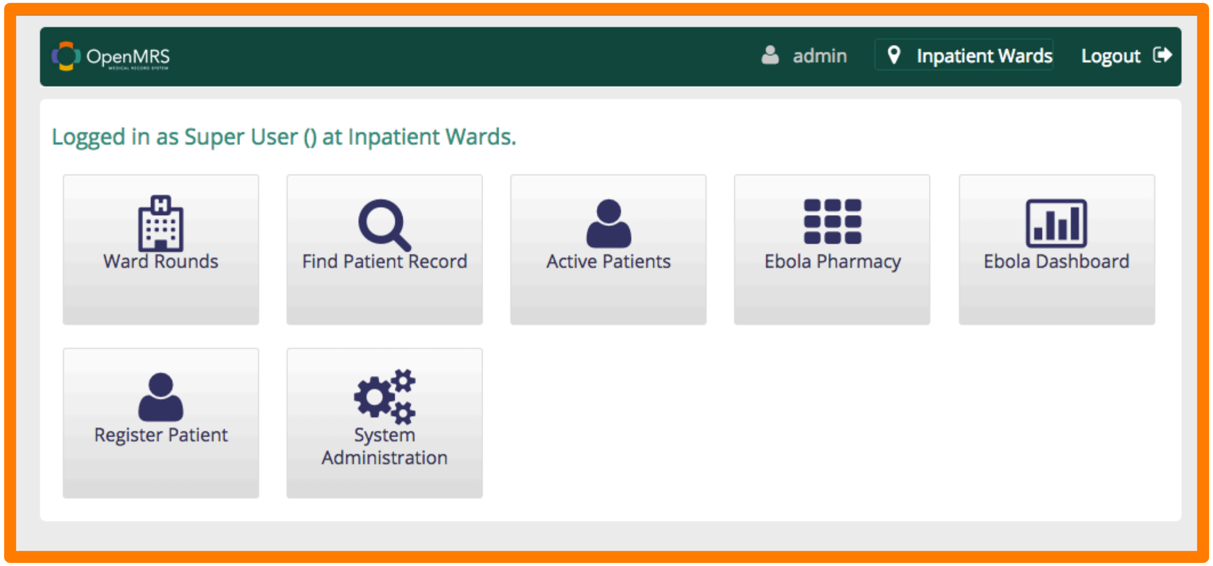

Desktop: patient registration

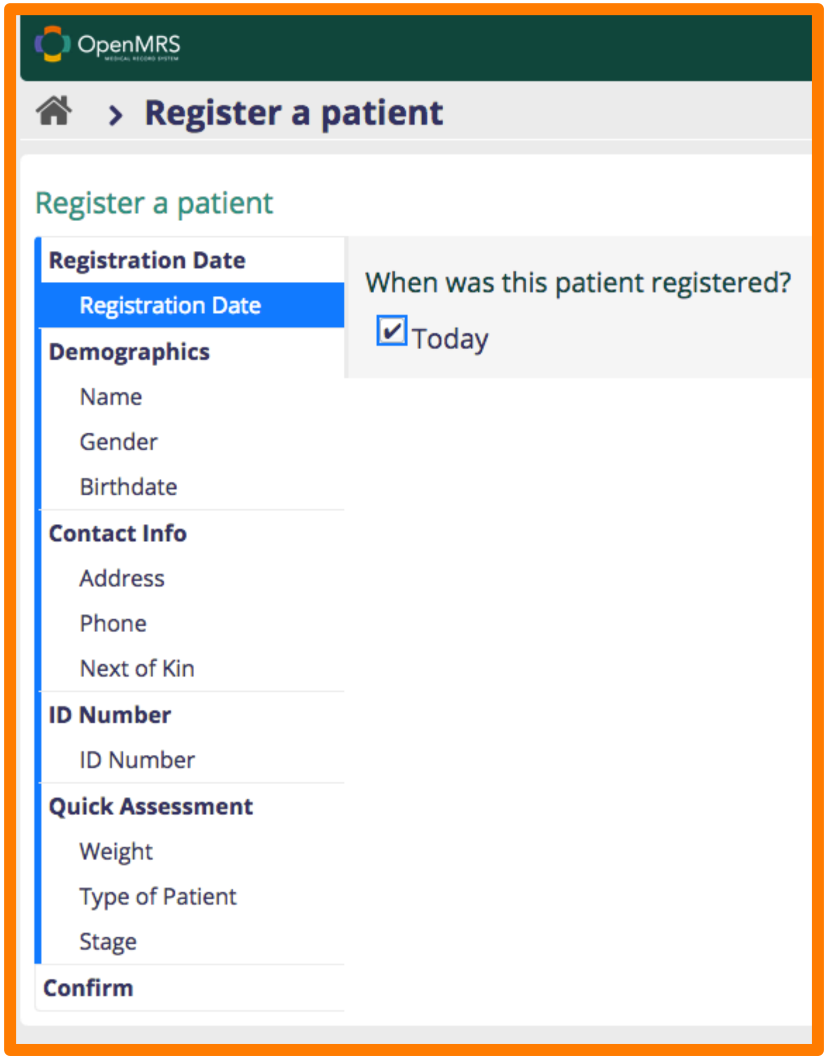

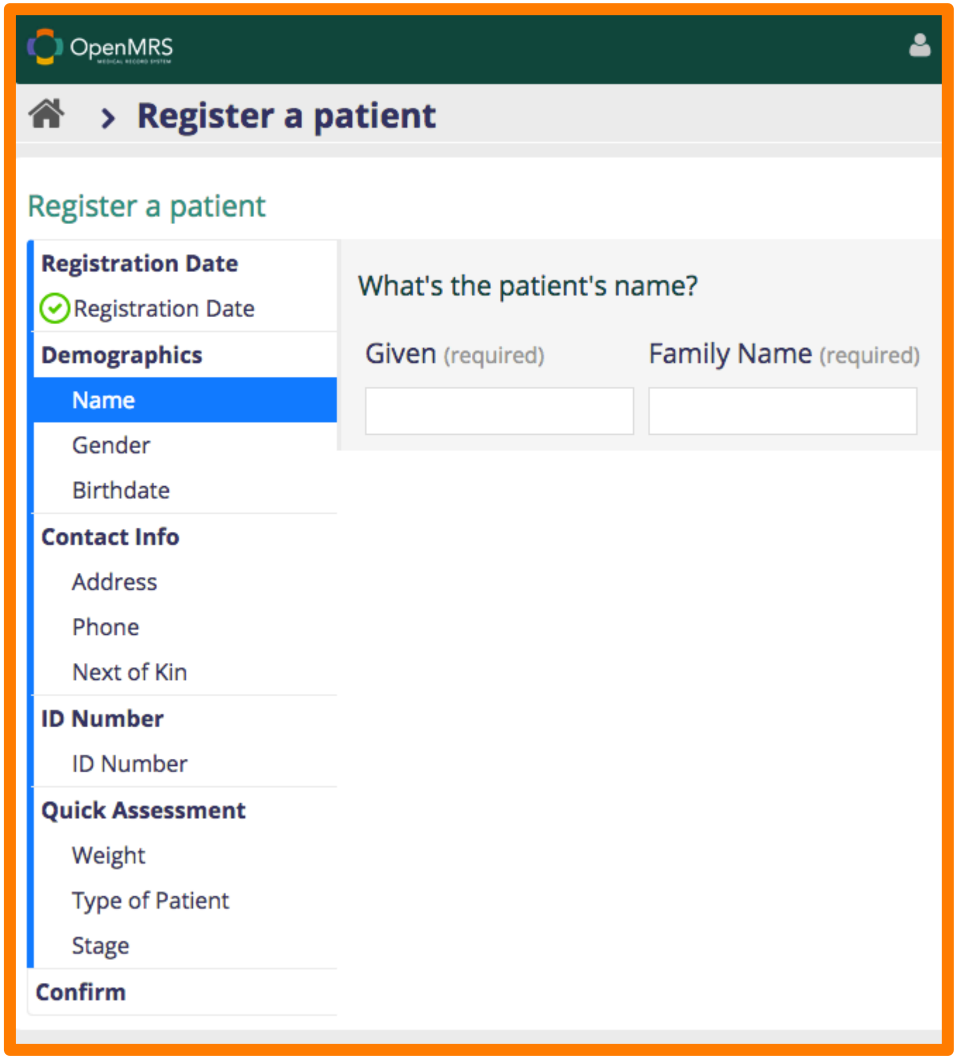

Desktop: patient registration

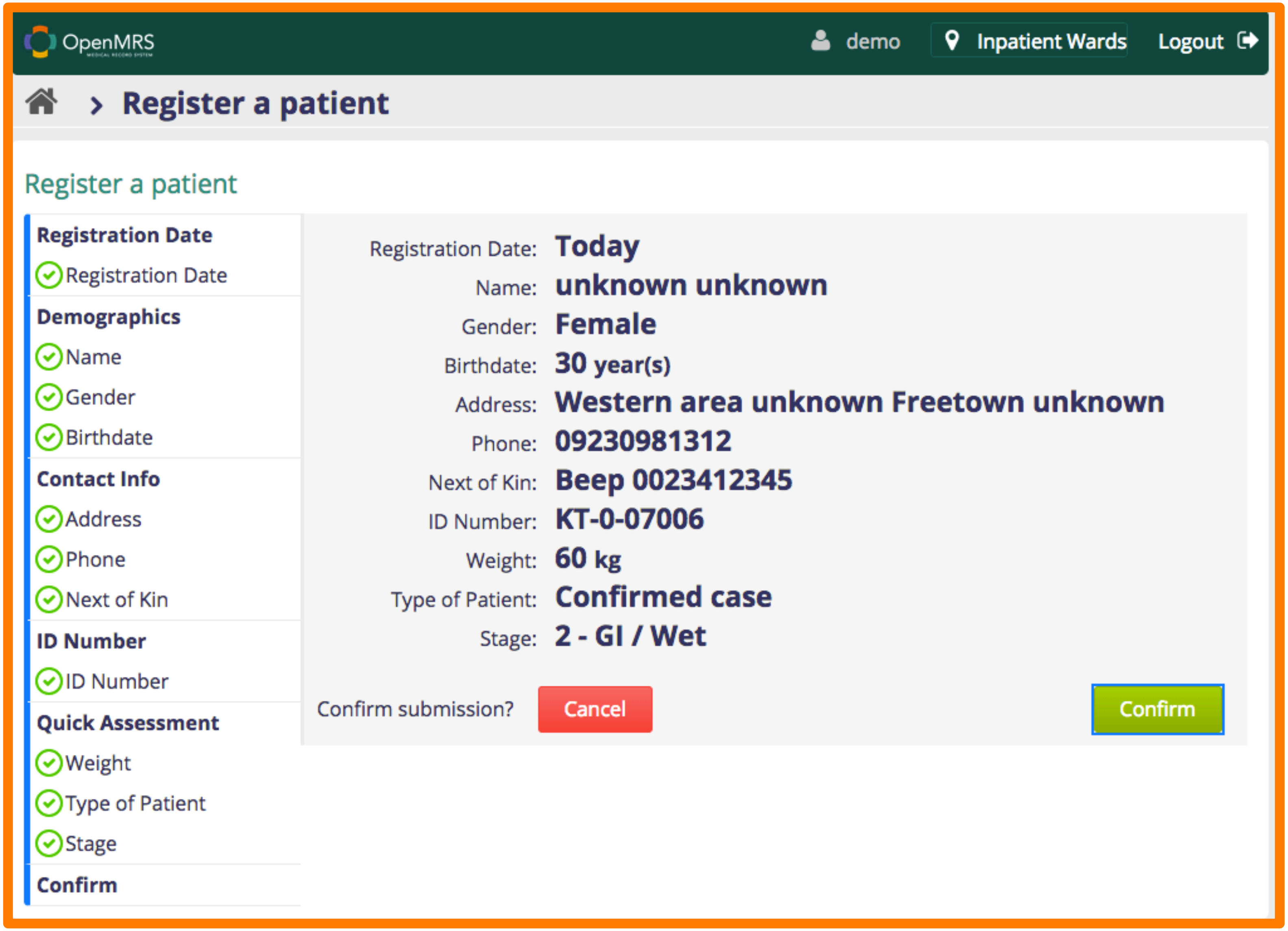

Desktop: bed allocation

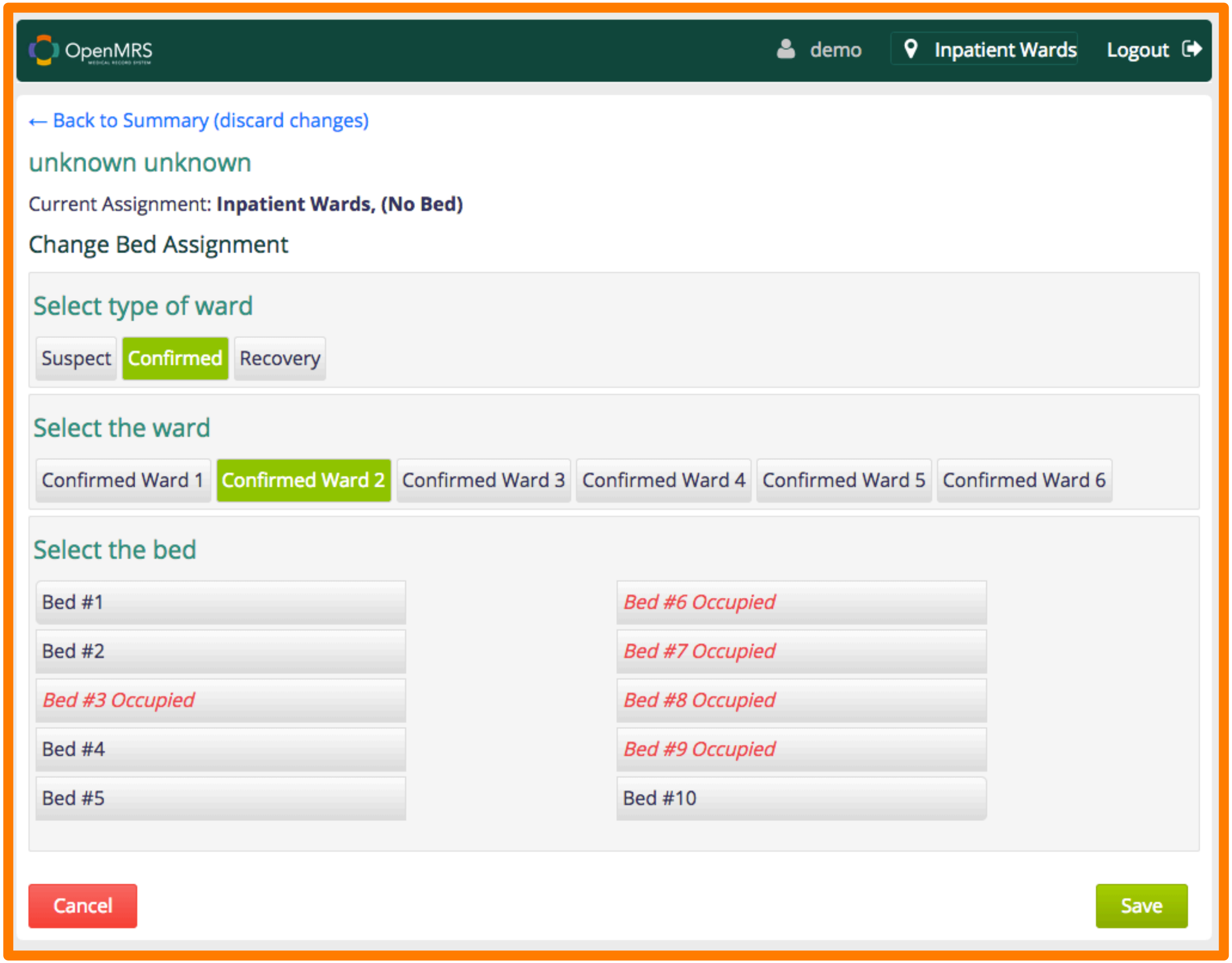

Desktop: active patients

OpenMRS admin Inpatient Wards Logout

Active Patients

View Active Patients: 07 Apr 2015

Confirmed Ward 1

Patient Number	Patient Name	Bed
KT-1-09876	Michael Pai	Bed #2
10027D	Leonardo Marquezini	Bed #3
KT-3-00031	Amani Tambo	Bed #6
KT-3-00021	Joram Mwesine	Bed #8
KT-3-00812	Romeo Bana	Bed #9

Confirmed Ward 2

Patient Number	Patient Name	Bed
KT-3-33333	John Doe	Bed #3
KT-3-45678	Happy Day	Bed #6
KT-3-99665	David Walton	Bed #7
KT-3-00024	Annet Sangaza	Bed #8
KT-0-22223	beep beepboop	Bed #9

Confirmed Ward 3

Patient Number	Patient Name	Bed
KT-9-55555	hylaeus alcyoneus	Bed #1

Confirmed Ward 5

Patient Number	Patient Name	Bed
KT-1-34568	fallacious mystic	Bed #10

Note: All patients and data included here are fictitious

Desktop: summary screens

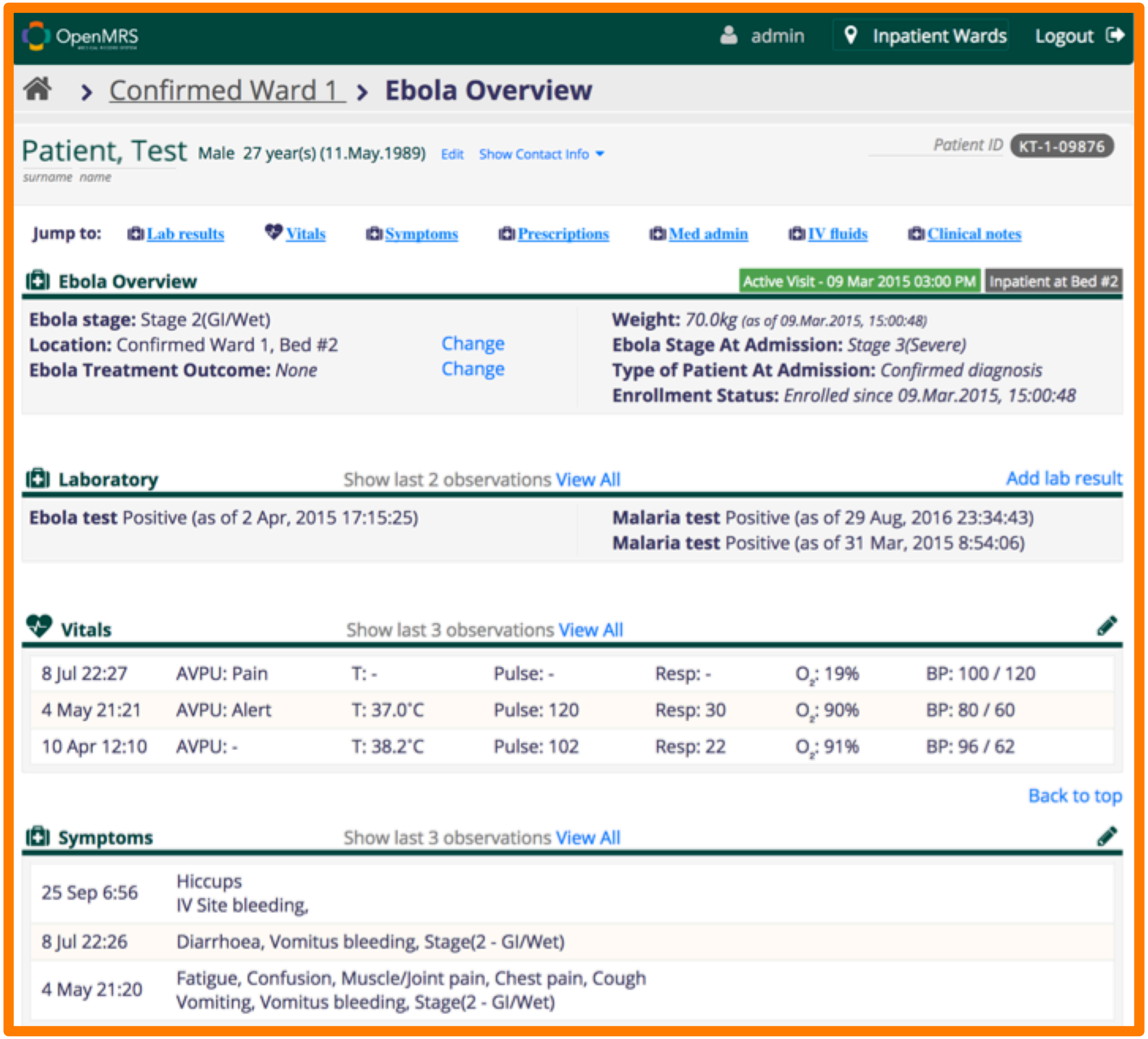

Desktop: summary screens

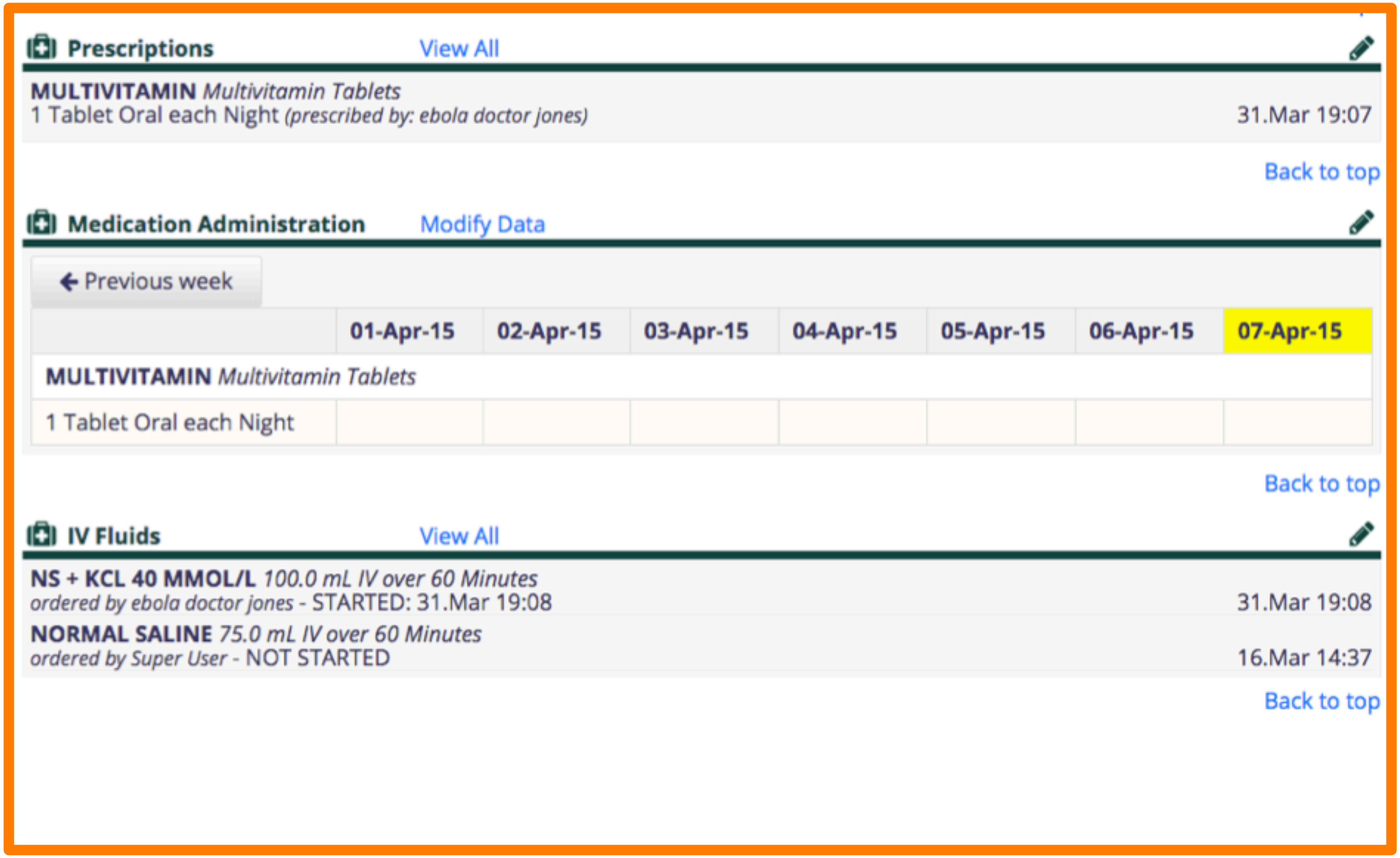

Desktop: drug administration charts

Medication Administration Modify Data

← Previous week

	01-Apr-15	02-Apr-15	03-Apr-15	04-Apr-15	05-Apr-15	06-Apr-15	07-Apr-15
**OMEPRAZOLE** *Omeprazole 20mg Capsule*							
2 Tablet Oral each Morning, Afternoon, Evening, Night PRN COUGH							START
**AMOXICILLIN** *Amoxicillin 500mg Capsules*							
1 Tablet Oral each Evening, Night for 4 Days							START
**ZINC SULFATE** *Zinc Sulfate 20mg Tablets*							
6 Microgram Oral each Morning			14:50 Fully Given				09:45 Fully Given<br>14:12 Not Given *Aggressive mood*<br>14:13 Fully Given

Desktop: lab results

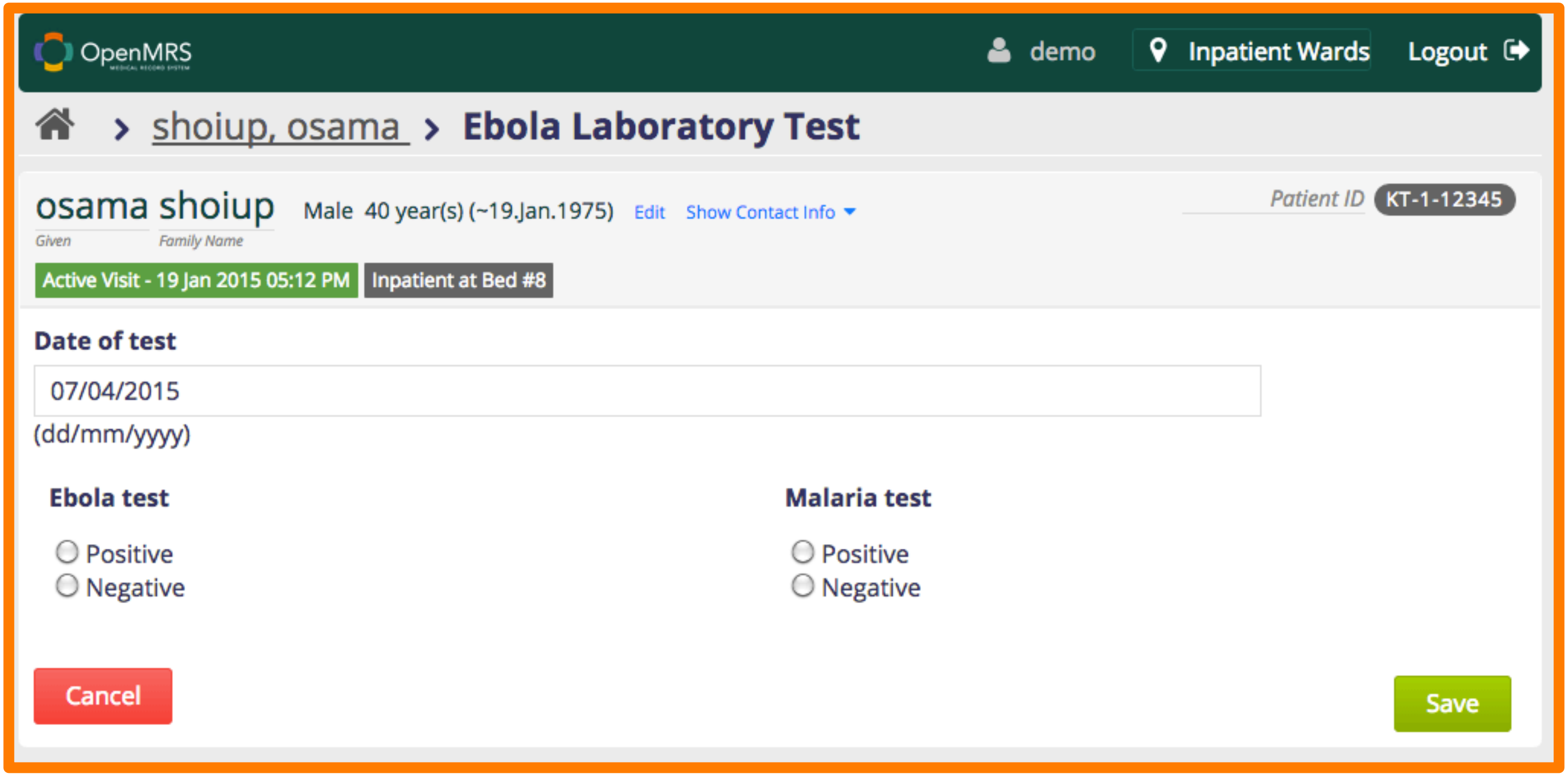

Desktop: clinical notes

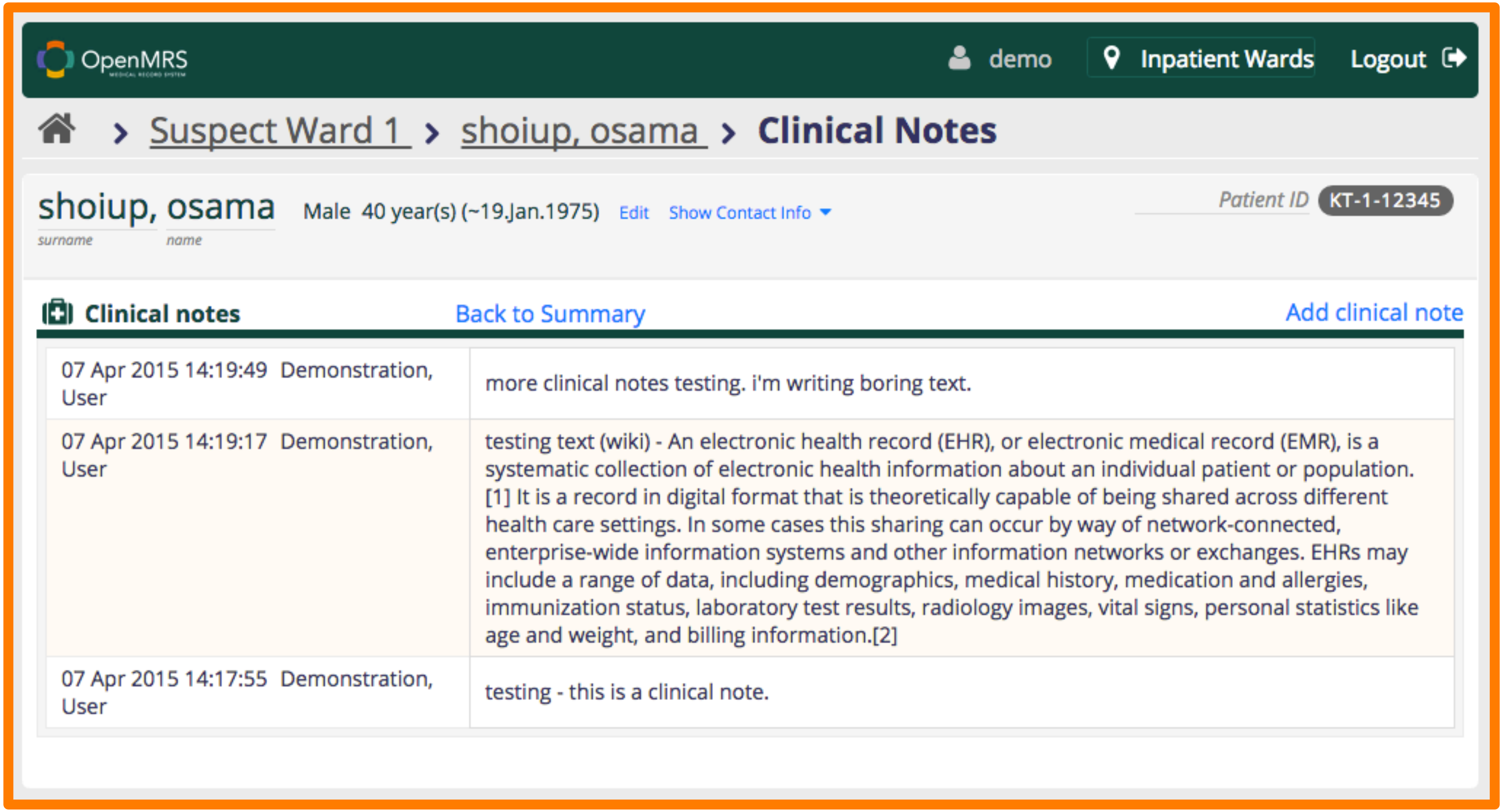

Desktop: discharge

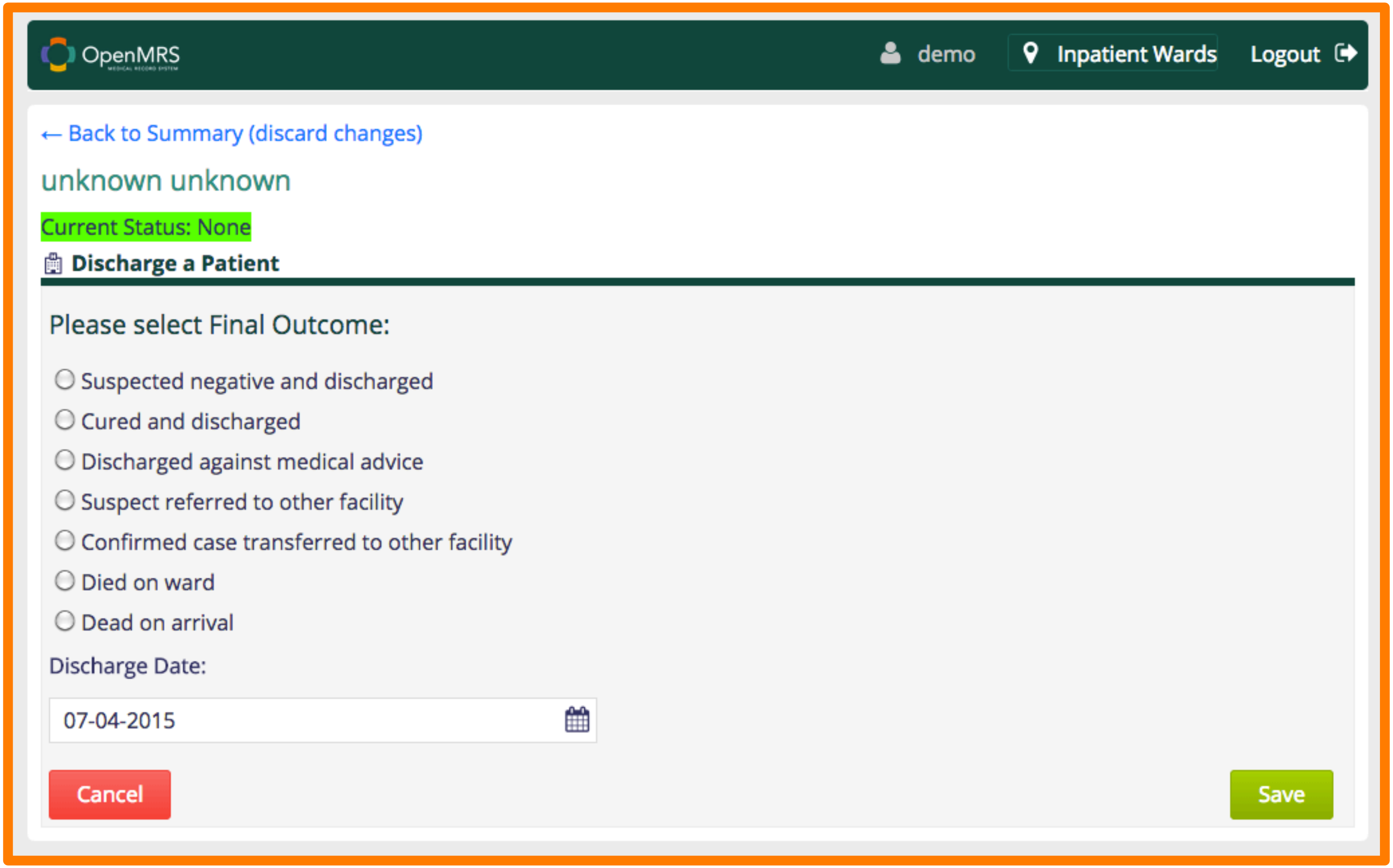

Supplement: Multimedia Appendix 1 [file jmir_v19i8e294_app1.pdf]
