# Supplementary material for: Development and Deployment of the OpenMRS-Ebola Electronic Health Record System for an Ebola Treatment Center in Sierra Leone
Source: J Med Internet Res. 2017 Aug 21;19(8):e294. doi: 10.2196/jmir.7881 (PMC5583502; doi:10.2196/jmir.7881)

Development and deployment of the OpenMRS-Ebola Electronic Health Record System for an Ebola Treatment Centre In Sierra Leone

Shefali Oza, Darius Jazayeri, Jonathan M. Teich, Ellen Ball, Patricia Nankubuge, Job Rwebembera, Kevin Wing, Alieu Sesay, Andrew S. Kanter, Glauber Ramos, David Walton, Rachael Cummings, Francesco Checchi, Hamish Fraser

Multimedia appendix 2:

Complete screenshots of the tablet-based OpenMRS-Ebola application

Primary use – Red (infectious) zone of Ebola Treatment Center

Note: All patients and data included here are fictitious

Tablet: team and ward selection

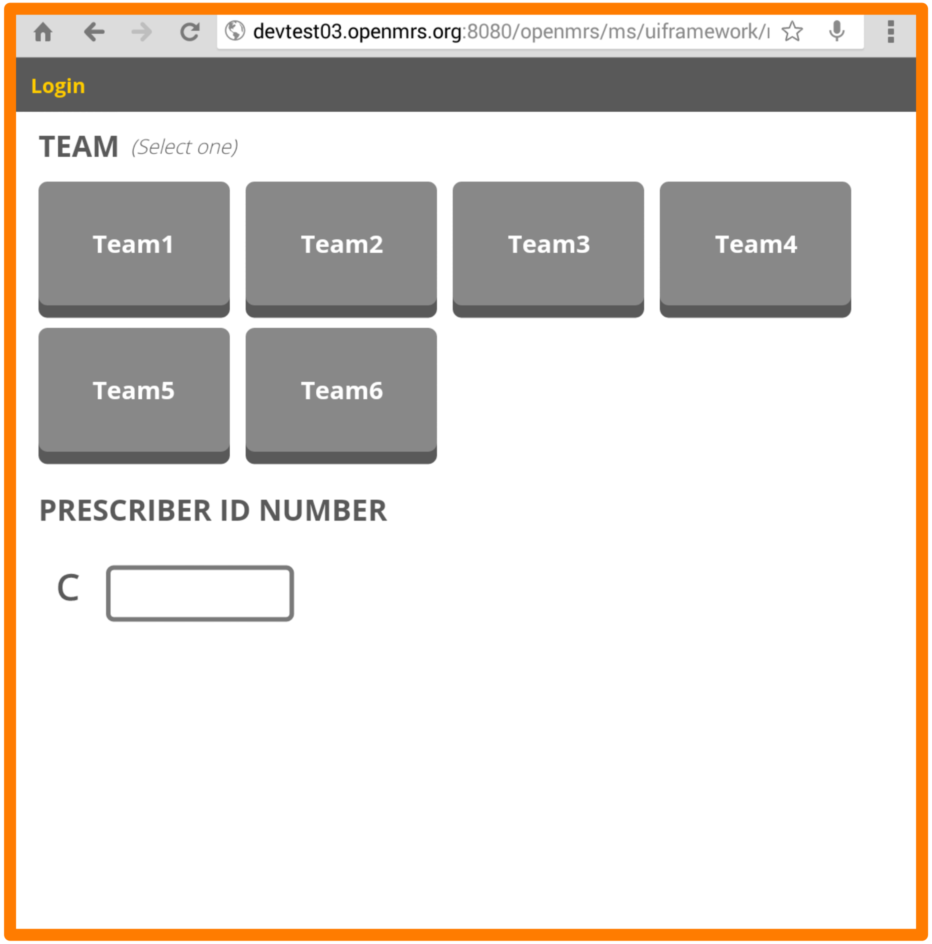

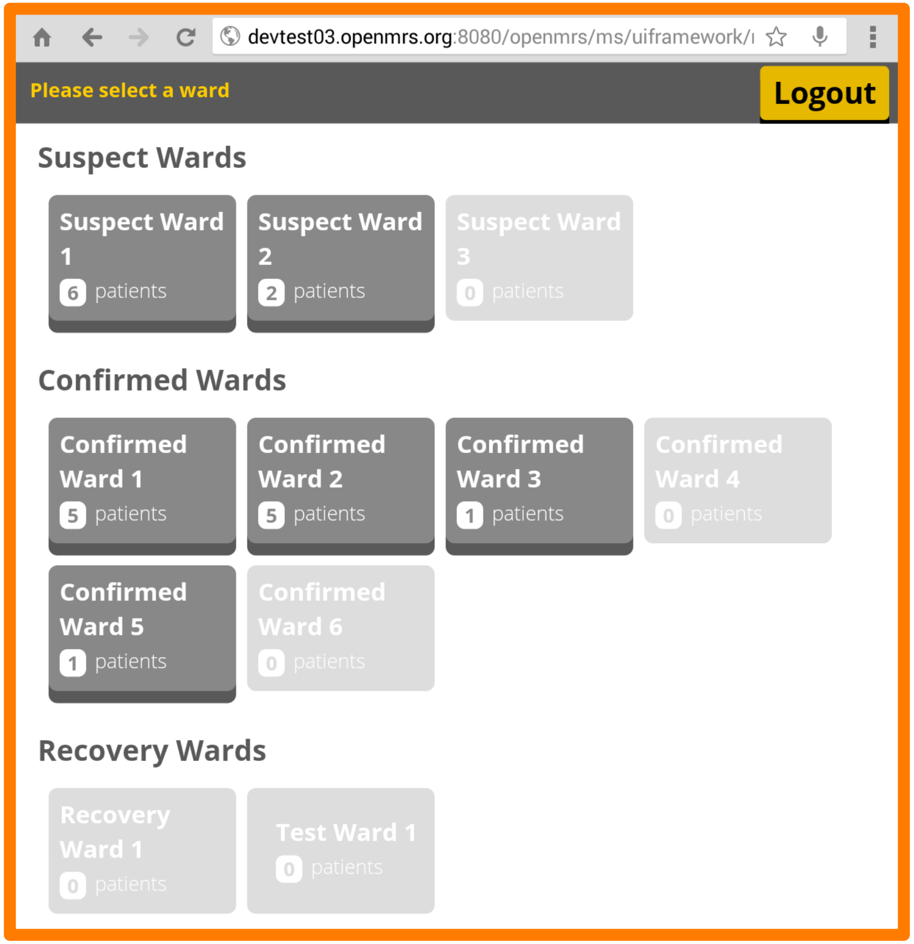

Tablet: patient selection and summary

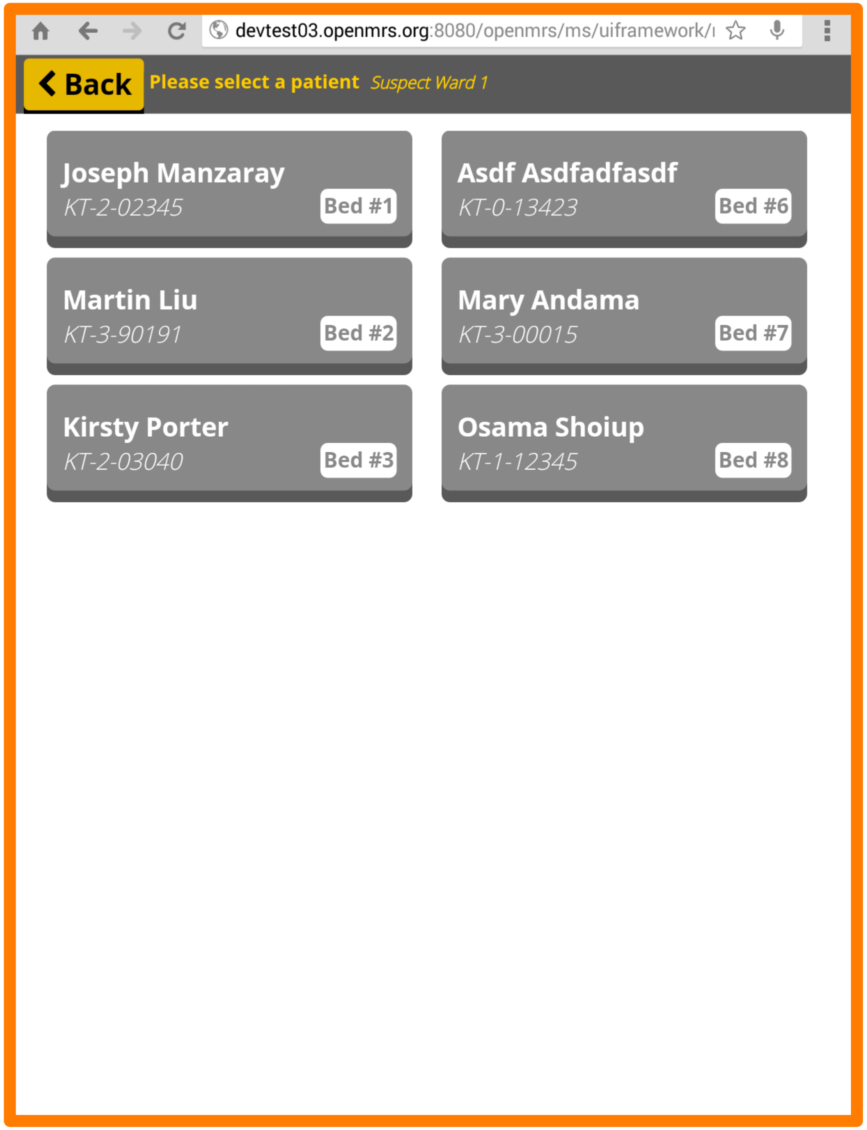

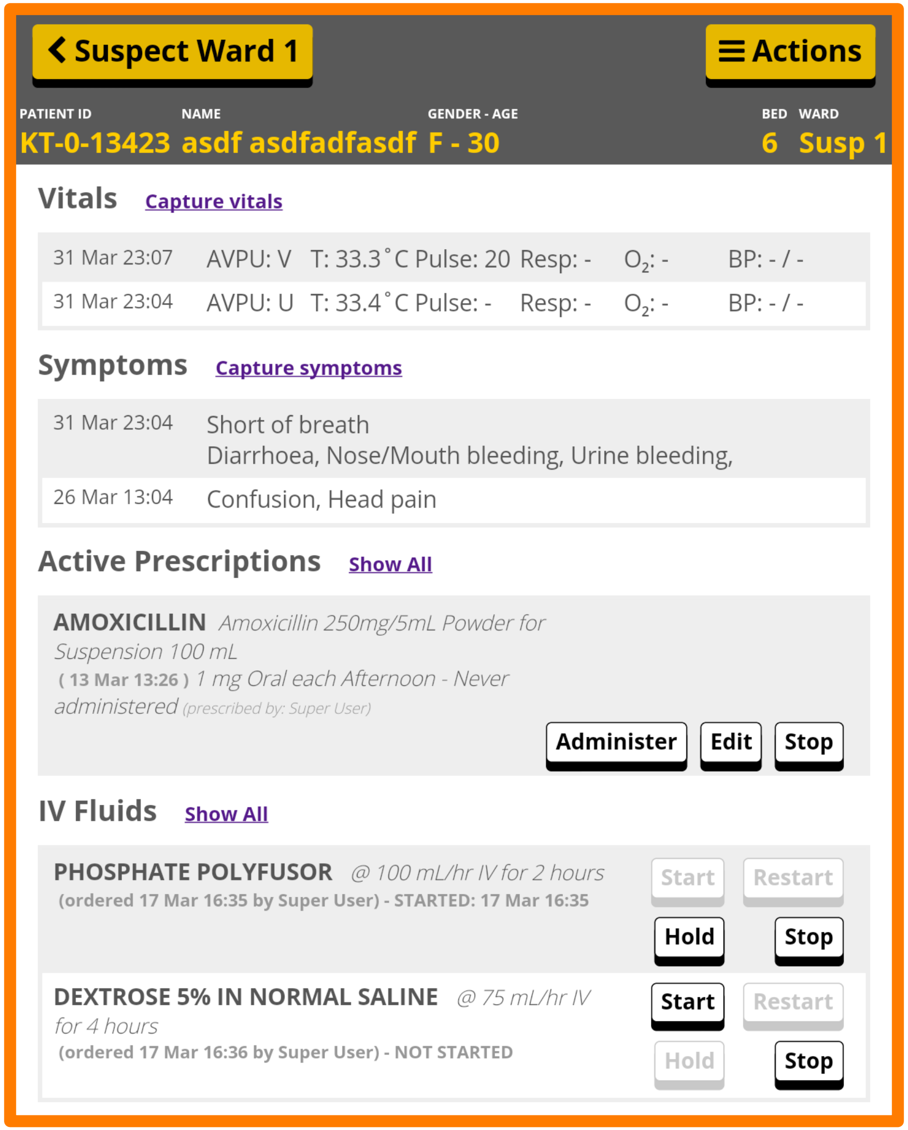

Note: All patients and data included here are fictitious

Tablet: action menu

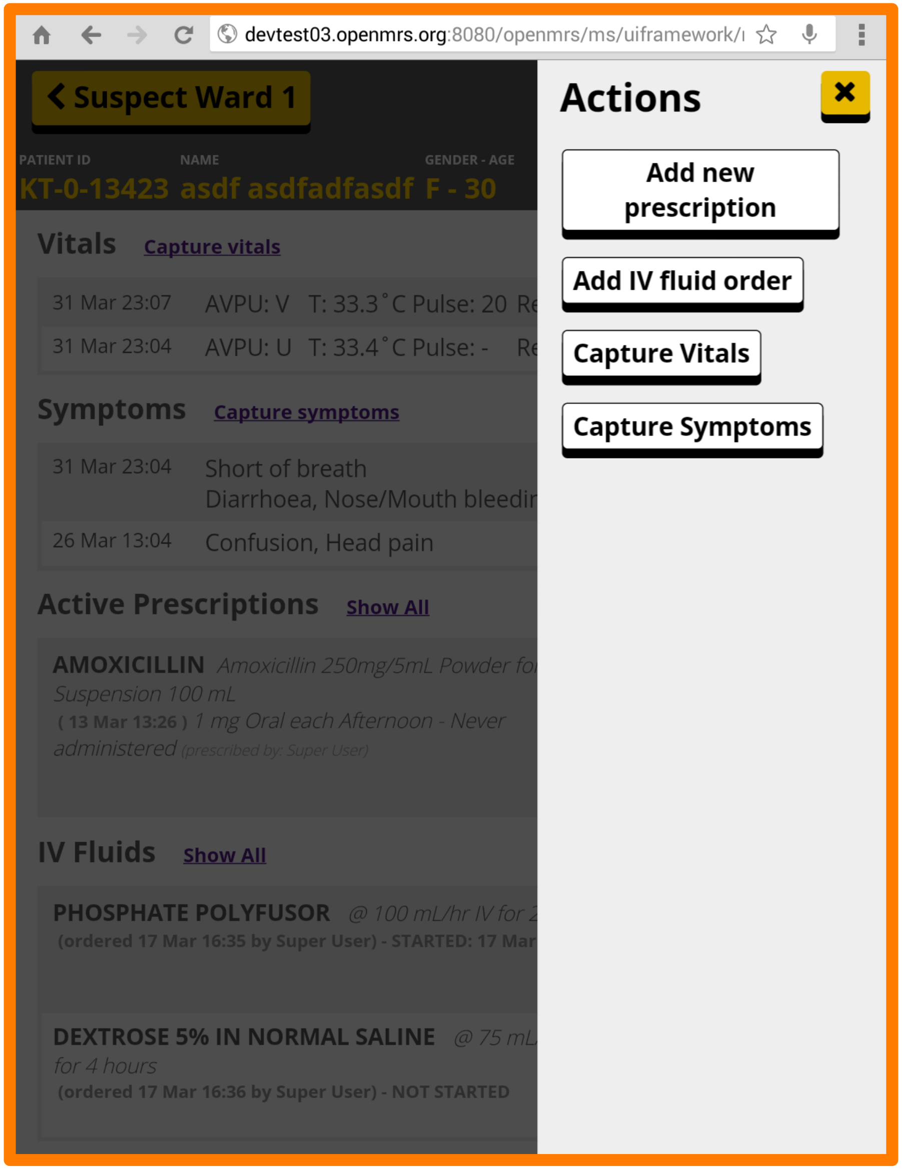

Tablet: drug selection

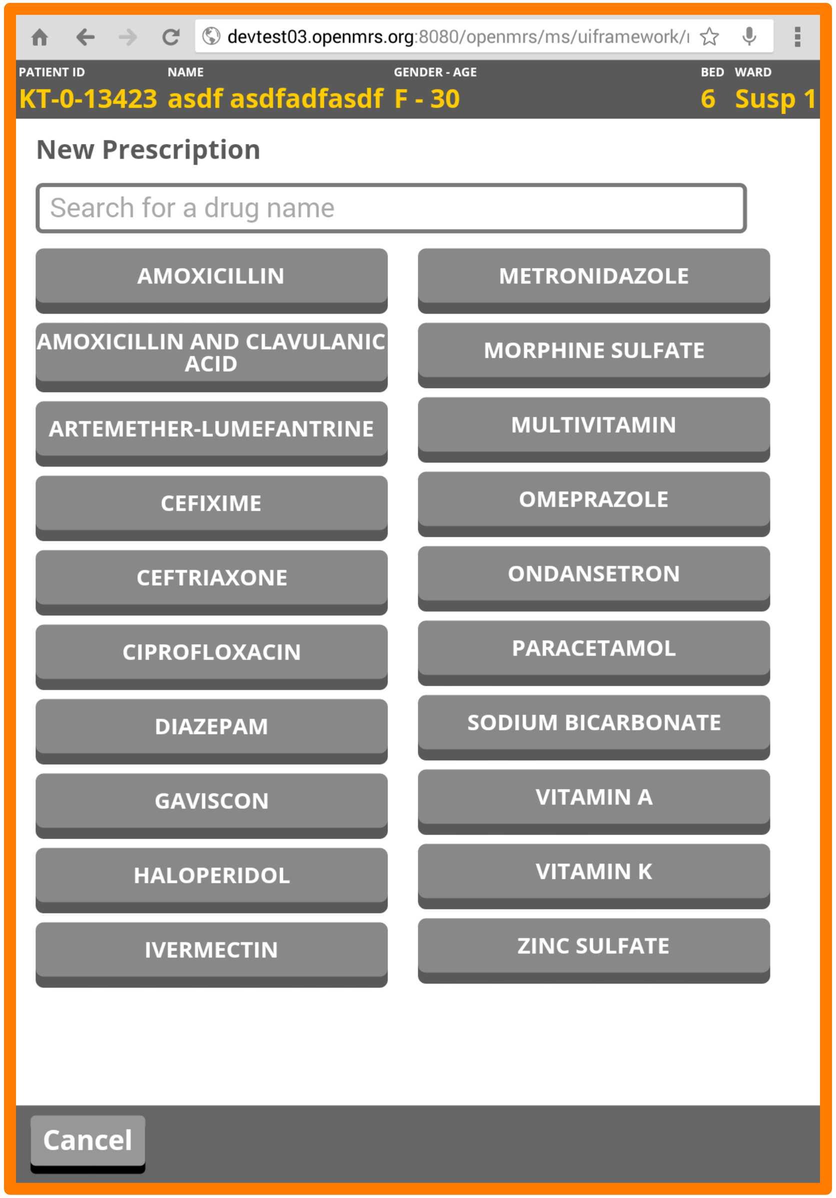

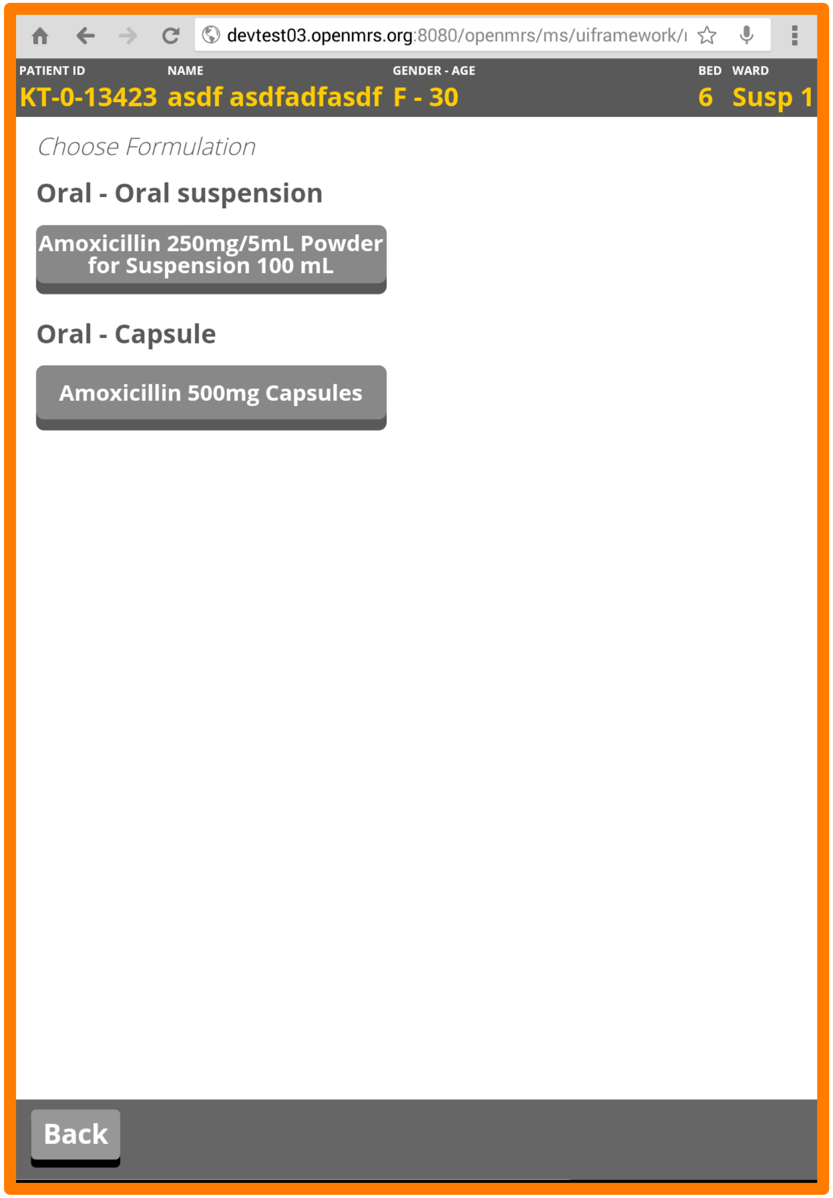

Tablet: drug details

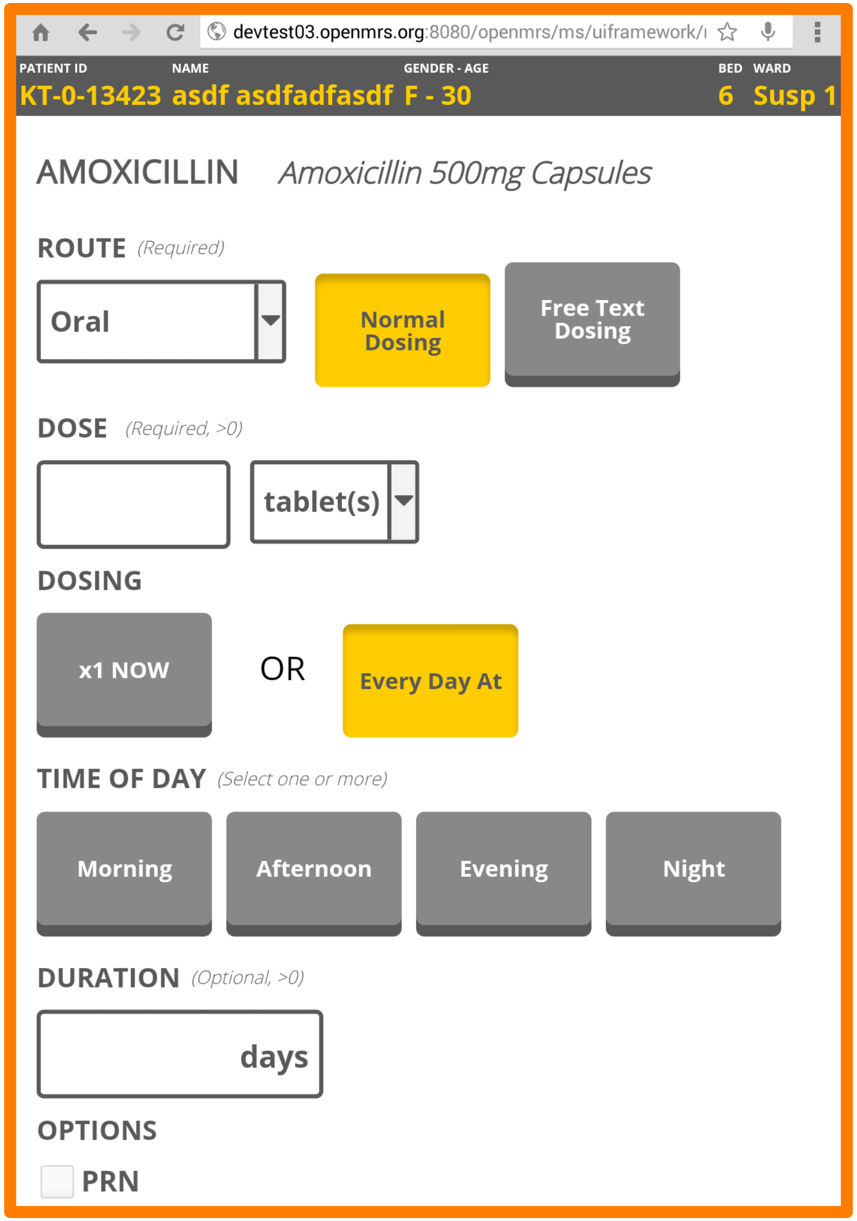

Tablet: drug administration

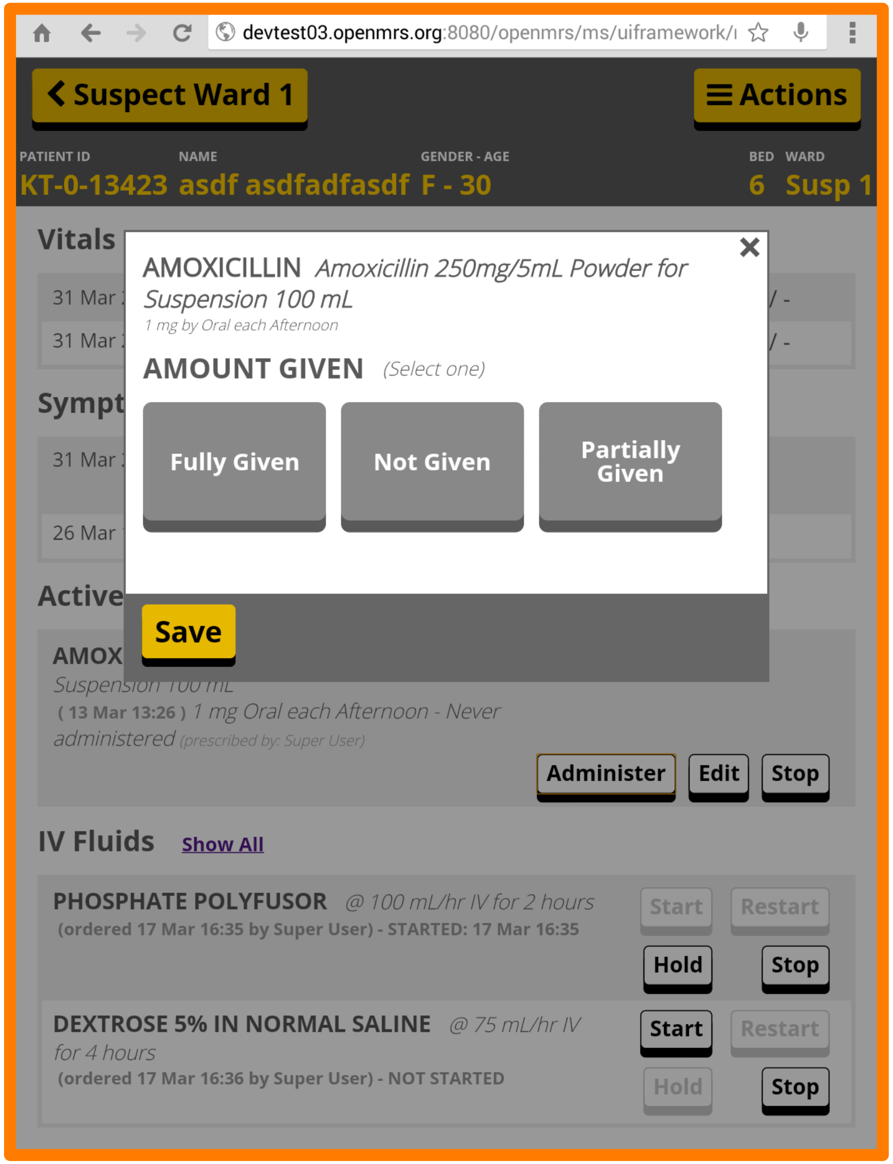

Tablet: IV fluid ordering

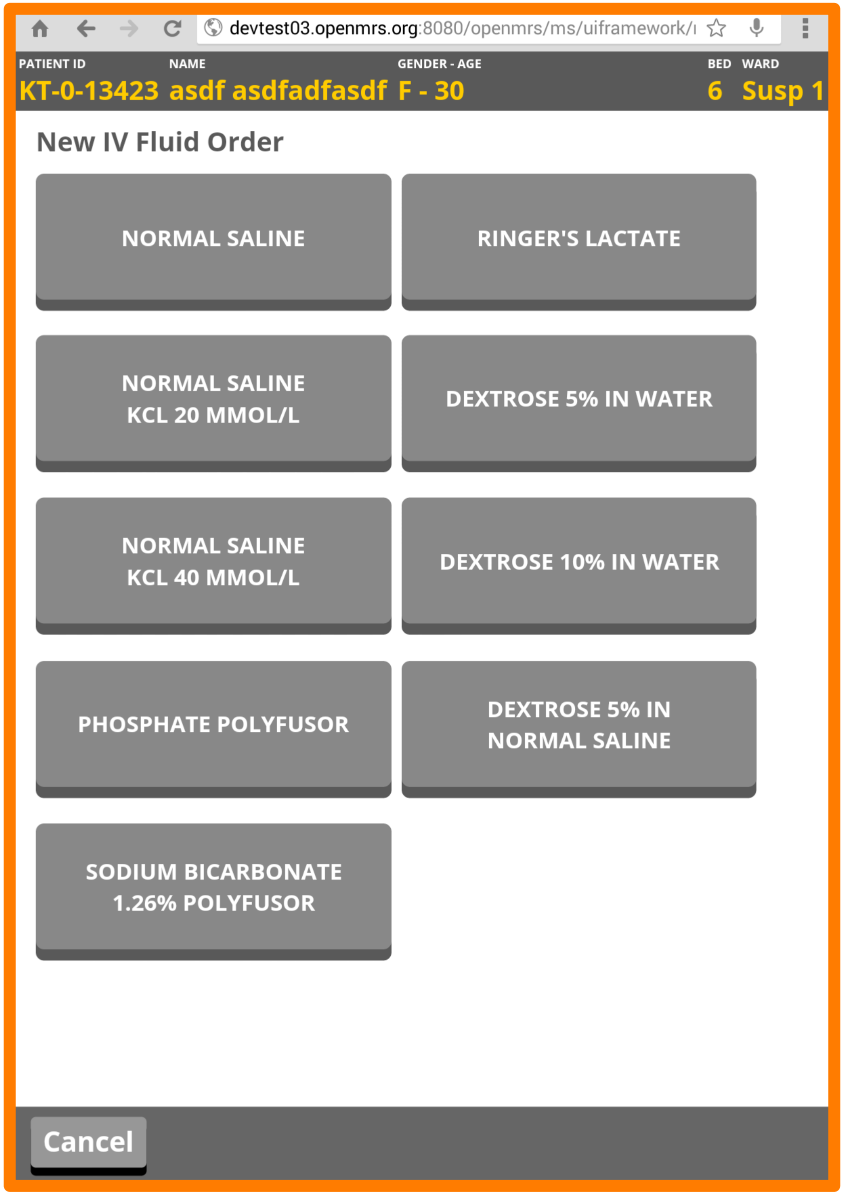

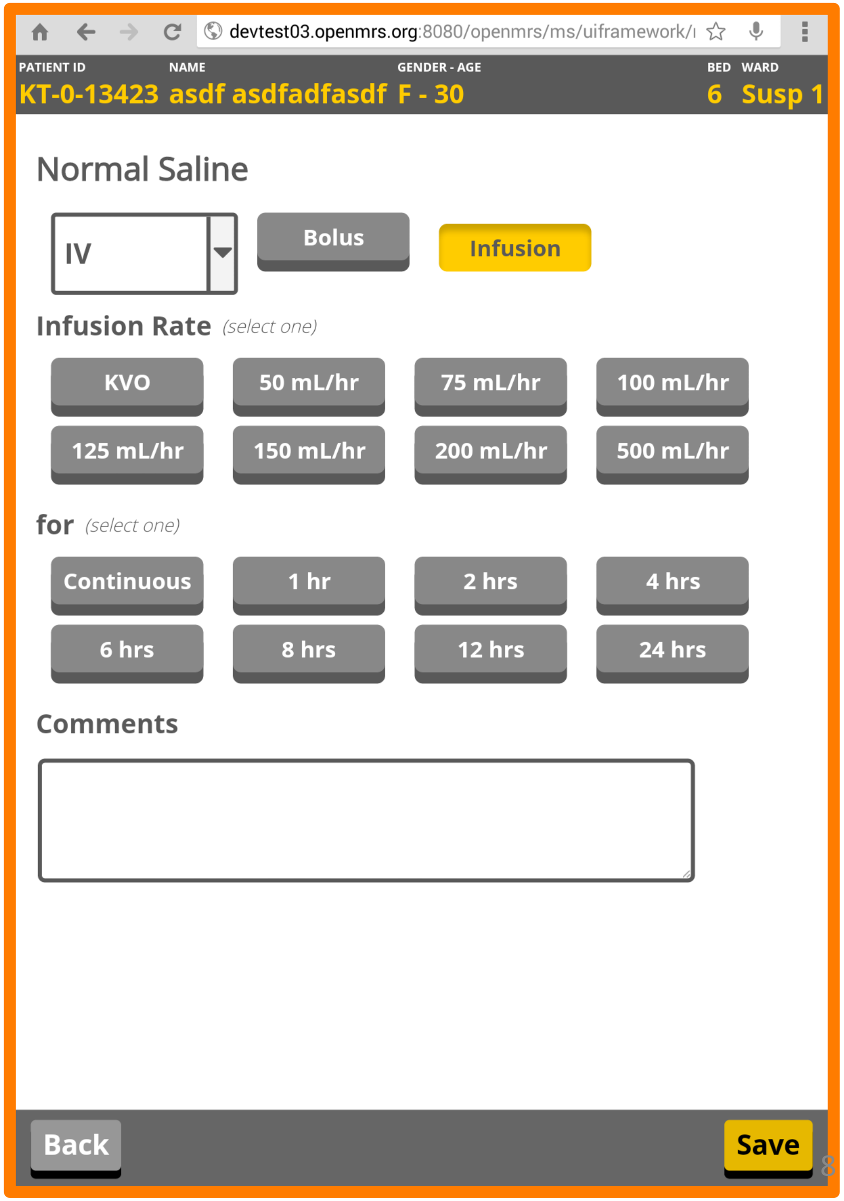

Tablet: IV fluid monitoring

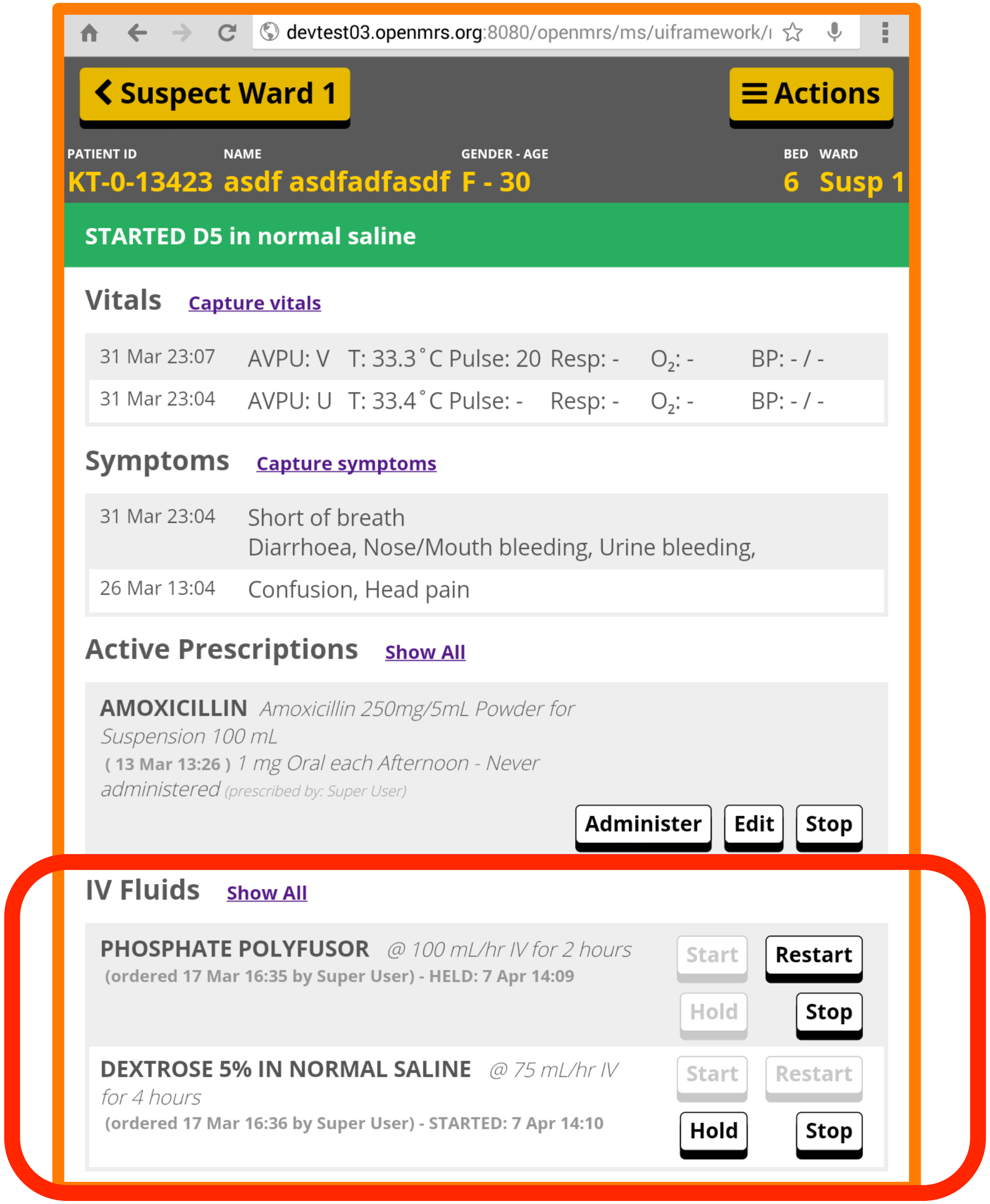

Tablet: vitals and symptoms

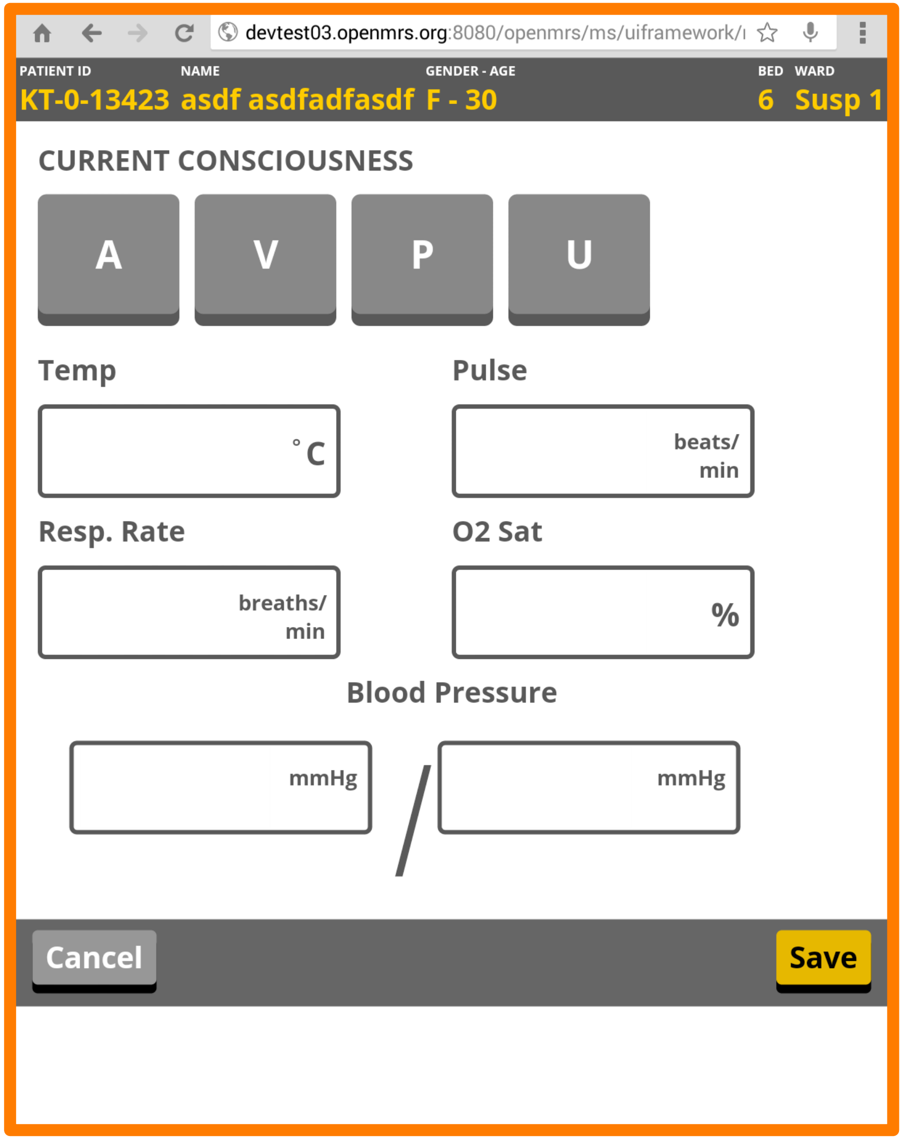

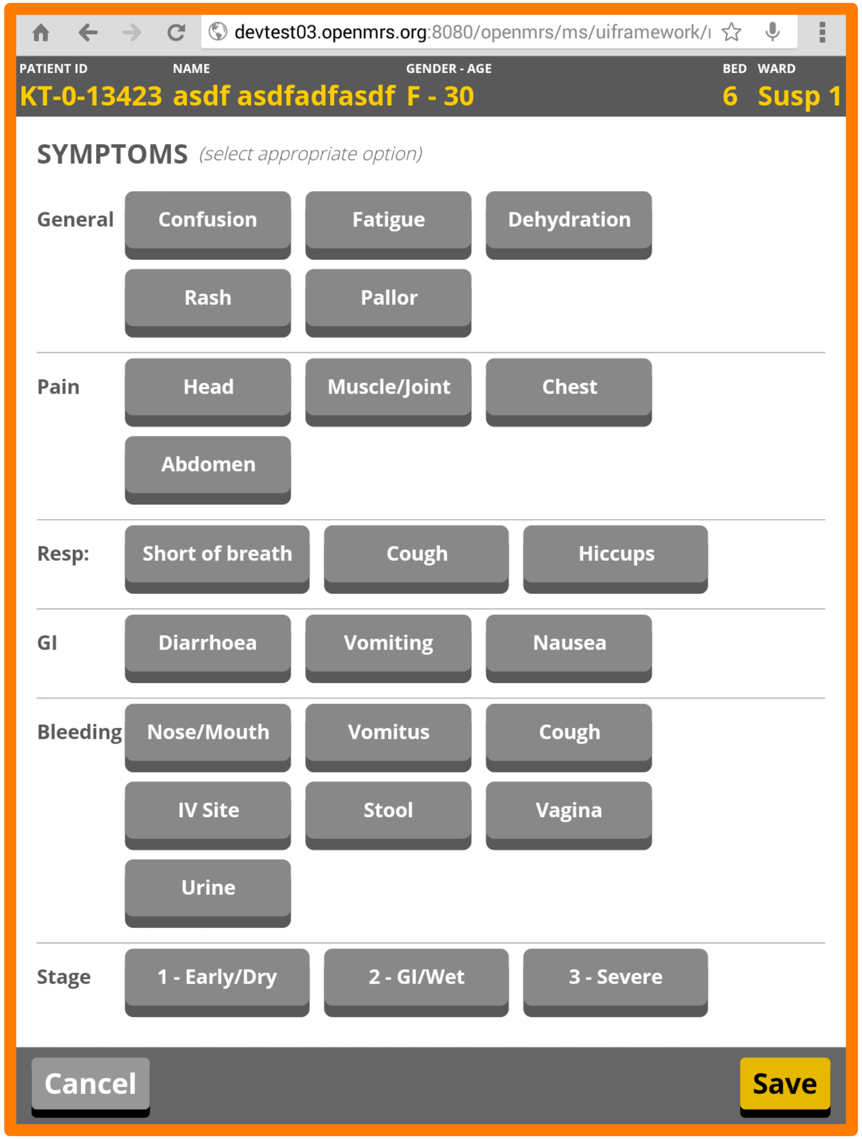

Supplement: Multimedia Appendix 2 [file jmir_v19i8e294_app2.pdf]
